# Plant Defense Activation by Endophytic *Metarhizium anisopliae* and *Beauveria bassiana* Fungi Against Subterranean Termites

**DOI:** 10.3390/ijms27093833

**Published:** 2026-04-25

**Authors:** Tanmaya Kumar Bhoi, Deepak Kumar Mahanta, Ipsita Samal, Sumit Jangra

**Affiliations:** 1 Forest Protection Division, ICFRE-Arid Forest Research Institute (ICFRE-AFRI), Jodhpur 342005, India; tkbhoi@icfre.org; 2 Forest Entomology Discipline, Forest Protection Division, Indian Council of Forestry Research and Education (ICFRE)-Forest Research Institute (ICFRE-FRI), Dehradun 248006, India; 3 ICAR-National Research Centre on Litchi, Mushahari, Ramna, Muzaffarpur 84202, India; happyipsu29@gmail.com; 4 UF/IFAS Tropical Research and Education Center, Homestead, FL 33031, USA

**Keywords:** endophytic entomopathogenic fungi, *Odontotermes obesus*, *Metarhizium anisopliae*, plant defense enzymes, termite biocontrol, sustainable forestry

## Abstract

Subterranean termites, particularly *Odontotermes obesus*, cause severe damage to forest nurseries and plantations in arid and semi-arid ecosystems. This study demonstrates the dual functional role of endophytic entomopathogenic fungi, *Metarhizium anisopliae* and *Beauveria bassiana*, in termite suppression and induction of plant defense responses. Laboratory bioassays revealed significantly higher virulence of *M. anisopliae*, with a lower LT_50_ (lethal time required to cause 50% mortality) of 33.1 h compared to *B. bassiana* (46.7 h), a steeper probit slope (5.4 ± 0.3), and strong model fit (R^2^ = 0.95), indicating rapid and synchronized mortality. Endophytic colonization varied across host species and application methods, with soil incorporation consistently outperforming foliar inoculation. Maximum colonization (82.5%) was recorded in *Tecomella undulata* and exceeded 80% in *Azadirachta indica* under *M. anisopliae*. Biochemical analyses revealed significant increases in protein (up to 3.5 mg g^−1^), phenols (3.7 mg g^−1^), and tannins (2.7 mg g^−1^). Activity of defense enzymes was significantly enhanced, with catalase reaching 263.5 U mL^−1^, while Phenylalanine ammonia-lyase and Tyrosine ammonia-lyase exceeded 170 and 198 U mL^−1^, respectively, indicating activation of antioxidant and phenylpropanoid pathways. Molecular docking analysis further revealed strong interactions between fungal metabolites and termite cellulase, with Bassianin (−8.4 kcal mol^−1^) and Tenellin (−8.1 kcal mol^−1^) showing the highest binding affinities. These findings highlight the combined biochemical and molecular mechanisms underlying fungal-mediated termite suppression and plant defense induction, and future research should prioritize transcriptomic validation, rhizosphere microbiome interactions, formulation optimization, and long-term multi-location field evaluation to support sustainable termite management strategies.

## 1. Introduction

Entomopathogenic fungi (EPF) are widely recognized as environmentally sustainable biological control agents capable of infecting and killing a broad range of insect pests [1,2,3,4]. Species such as *Beauveria bassiana* and *Metarhizium anisopliae* infect insects through cuticular penetration, internal colonization, and the production of insecticidal metabolites, ultimately leading to host mortality [3,4,5,6]. Although these fungi have demonstrated strong pathogenicity under laboratory conditions, their field performance against soil-dwelling insects often remains inconsistent [7]. Environmental stresses, microbial competition, and host defense behaviors can limit fungal persistence and transmission in natural settings. Nevertheless, recent advances have revealed an additional ecological role of EPF beyond direct pathogenicity, their ability to establish as endophytes within plant tissues [7,8].

In their endophytic state, entomopathogenic fungi colonize internal plant tissues without causing disease, forming stable and often mutualistic associations with the host [9]. This internal colonization protects fungal propagules from adverse environmental conditions and enables long-term persistence within plant systems [10]. More importantly, endophytic entomopathogenic fungi (EEPF) can modulate plant physiological and biochemical pathways associated with defense and stress tolerance. Thus, EEPF provide a dual functional benefit: they act as internal reservoirs of insect-pathogenic potential and simultaneously enhance plant defensive capacity [9,10,11].

Activation of plant defense mechanisms by endophytic colonization involves modulation of both primary and secondary metabolic pathways [12,13,14]. Colonized plants frequently exhibit increased levels of phenolic compounds and tannins, secondary metabolites known to deter herbivores and inhibit microbial invasion [15]. Central to this response are enzymes of the phenylpropanoid pathway, particularly phenylalanine ammonia-lyase (PAL) and tyrosine ammonia-lyase (TAL), which regulate the biosynthesis of phenolic defenses [16]. Additionally, antioxidant enzymes such as ascorbate oxidase (AO) help regulate reactive oxygen species generated during biotic stress, thereby maintaining cellular integrity [2,3,4]. Enhanced total protein content in colonized plants may further reflect improved metabolic activity and resilience under pest pressure. Collectively, these biochemical modifications suggest that EEPF can prime or activate systemic defense responses in host plants [6].

Subterranean termites, especially *Odontotermes obesus* (Rambur, 1842), are among the most destructive soil-dwelling pests in agroforestry systems and forest nurseries [2,17,18]. Feeding primarily on cellulose-rich tissues, these termites attack roots, seedlings, and young plantations below ground, where damage remains undetected until severe plant mortality occurs. In arid and semi-arid regions, where tree regeneration is already constrained by harsh environmental conditions, termite infestation significantly reduces seedling establishment and plantation productivity [3,4]. The cryptic habitat and highly organized social structure of subterranean termites, including grooming and removal of infected individuals, make their management particularly challenging [5,6].

Conventional termite control has relied largely on synthetic termiticides. While effective in the short term, repeated chemical applications have resulted in soil and groundwater contamination, disruption of beneficial soil microorganisms, and risks to non-target organisms [19,20]. Furthermore, chemical treatments often fail to provide durable protection in plantation systems because termite colonies can reinvade treated sites from adjacent untreated areas. These ecological and operational limitations underscore the need for integrated, sustainable alternatives [21,22].

Although EPF have demonstrated pathogenicity against termites, their success in field conditions is often limited by termite social immunity and environmental degradation of fungal propagules [2,3]. Endophytic colonization offers a promising strategy to overcome these constraints by positioning the fungus within plant tissues, where it may directly or indirectly affect termite feeding and survival [4,5,6]. Changes in plant biochemical composition induced by EEPF, such as elevated phenols, tannins, and defense-related enzymes, could alter tissue palatability, reduce digestibility, or interfere with termite physiology and symbiotic digestion [7]. However, the mechanisms by which endophytic EPF activate plant defense responses against subterranean termites remain insufficiently explored.

The present study was therefore designed to investigate defense activation in forest plant species colonized by endophytic entomopathogenic fungi with biocontrol potential against *O. obesus*. We hypothesized that plants colonized by *B. bassiana* and *M. anisopliae* would exhibit enhanced resistance to termite attack through a combination of direct fungal pathogenic effects and fungus-mediated activation of plant biochemical defenses. Specifically, this research aimed to (i) evaluate the virulence of selected EPF isolates against worker termites, (ii) assess endophytic colonization of selected forest plant species by EEPF, (iii) evaluate their biocontrol efficacy against *O. obesus*, (iv) quantify changes in key biochemical defense indicators, including ascorbate oxidase (AO), phenylalanine ammonia-lyase (PAL), tyrosine ammonia-lyase (TAL), total phenols, tannins, and total protein, and (v) investigate the interaction of selected fungal secondary metabolites with termite cellulase through molecular docking analysis. By integrating fungal endophytism, plant defense activation, and computational screening of fungal metabolites, this study aims to develop a scientifically robust and environmentally sustainable strategy for termite management in forest nurseries and plantation ecosystems.

## 2. Results

### 2.1. Virulence of Entomopathogenic Fungi Against O. obesus

*M. anisopliae* exhibited significantly higher efficacy, causing faster and more uniform mortality in the termite population. *M. anisopliae* recorded a lower LT_50_ value of 33.12 h, with narrow 95% fiducial limits (FL) ranging from 31.75 to 34.62 h, indicating a rapid onset of pathogenic action (Table 1 and Table 2). This high virulence is further supported by a steep slope value (5.38 ± 0.27), reflecting a strong and synchronized mortality response, along with a low heterogeneity factor (h = 0.46), suggesting minimal variability among treated individuals. The LT_90_ value of 60.84 h (FL: 56.73–65.91) confirms the ability of *M. anisopliae* to achieve high levels of mortality within a relatively short exposure period. The goodness-of-fit of the probit model was satisfactory (χ^2^ = 1.42, *p* = 0.7), with a high coefficient of determination (R^2^ = 0.95), validating the reliability of the estimates. *B. bassiana* exhibited a slower and less consistent pathogenic effect. The LT_50_ value was comparatively higher at 46.78 h (FL: 41.92–51.80), indicating delayed mortality, while the LT_90_ value increased substantially to 101.42 h with wide fiducial limits (FL: 80.35–148.26), reflecting greater variability in response. The slope value (4.01 ± 0.25) was lower than that of *M. anisopliae*, and the heterogeneity factor was notably higher (h = 2.31), suggesting heterogeneous susceptibility within the termite population. Although the probit model fit was acceptable (χ^2^ = 7.35, *p* = 0.1; R^2^ = 0.93), the broader FL width (59.5 h) indicates less precision in mortality prediction.

### 2.2. Effect of Inoculation Method on Endophytic Colonization of Entomopathogenic Fungi in Selected Forest Tree Species

Endophytic colonization varied significantly among fungal species and inoculation methods across all tree species. *M. anisopliae* consistently showed higher colonization than *B. bassiana*. In *T. grandis*, colonization by *M. anisopliae* reached 62.3 ± 2.5% (foliar) and 78.4 ± 2.8% (soil), compared to 33.1 ± 1.8% and 49.3 ± 2.0% for *B. bassiana*. Similar trends were observed in *P. pinnata* (60.8 ± 2.4% and 75.2 ± 2.7% vs. 31.5 ± 1.7% and 46.1 ± 1.9%), *D. sissoo* (61.2 ± 2.5% and 76.5 ± 2.6% vs. 29.8 ± 1.5% and 44.8 ± 1.8%), and *A. indica* (63.0 ± 2.6% and 80.1 ± 2.9% vs. 31.5 ± 1.6% and 46.5 ± 1.9%). In *S. senegal* and *P. cineraria*, *M. anisopliae* exceeded 60% (foliar) and 74% (soil), whereas *B. bassiana* remained below 50%. The highest colonization was recorded in *T. undulata* (65.2 ± 2.7% foliar; 82.5 ± 3.0% soil for *M. anisopliae* vs. 33.6 ± 1.8% and 48.2 ± 2.0% for *B. bassiana*). Across all species, soil incorporation resulted in significantly higher colonization than foliar application. Across all tree species evaluated, soil incorporation resulted in significantly greater colonization than foliar spray for both fungal species. *M. anisopliae* demonstrated significantly superior endophytic establishment compared to *B. bassiana*, irrespective of host species or inoculation method (Supplementary Figure S1a,b).

### 2.3. Comparative Influence of Foliar Spray and Soil Application on Tissue-Specific Endophytic Colonization

Endophytic colonization varied significantly between fungal species and inoculation methods across all tree species. Following foliar inoculation, *M. anisopliae* consistently exhibited higher colonization than *B. bassiana* across tissues. In *T. grandis*, colonization was 58.2%, 61.5%, and 67.2% (roots, stems, leaves) versus 28.1%, 32.4%, and 38.8%, respectively. Similar trends were observed in *P. pinnata* (56.8%, 60.2%, 65.3% vs. 27.5%, 30.2%, 33.8%), *D. sissoo* (57.5%, 60.8%, 65.3% vs. 26.8%, 30.1%, 32.5%), and *A. indica* (59.2%, 62.5%, 67.5% vs. 28.2%, 31.5%, 35.3%). In *S. senegal* and *P. cineraria*, *M. anisopliae* exceeded 55% (roots) and 62% (leaves), whereas *B. bassiana* remained below 33%. The highest foliar colonization occurred in *T. undulata* (61.8%, 64.5%, 69.2% vs. 30.2%, 33.6%, 38.0%), with slightly higher colonization in leaves (Supplementary Figure S2a–c). Soil incorporation significantly enhanced colonization. In *T. grandis*, *M. anisopliae* reached 87.4%, 75.2%, and 72.5% compared to 55.3%, 50.2%, and 48.3% for *B. bassiana*. Comparable patterns were observed in *P. pinnata* (84.5%, 73.0%, 70.2% vs. 53.0%, 48.0%, 46.1%), *D. sissoo* (85.3%, 74.0%, 71.5% vs. 52.7%, 47.0%, 44.8%), and *A. indica* (87.5%, 75.5%, 73.0% vs. 53.0%, 48.2%, 46.5%). In *S. senegal* and *P. cineraria*, root colonization by *M. anisopliae* exceeded 83%, whereas *B. bassiana* remained below 52%. *T. undulata* again showed the highest colonization (87.5% roots; 75.2% leaves vs. 55.5% and 48.2%). Soil inoculation resulted in greater colonization than foliar application, and *M. anisopliae* demonstrated superior systemic establishment across all hosts and tissues (Supplementary Figure S2d–f).

### 2.4. Variation in Protein, Phenol, and Tannin Content in Tree Seedlings Following Endophytic Colonization by Entomopathogenic Fungi

**Figure 1:**
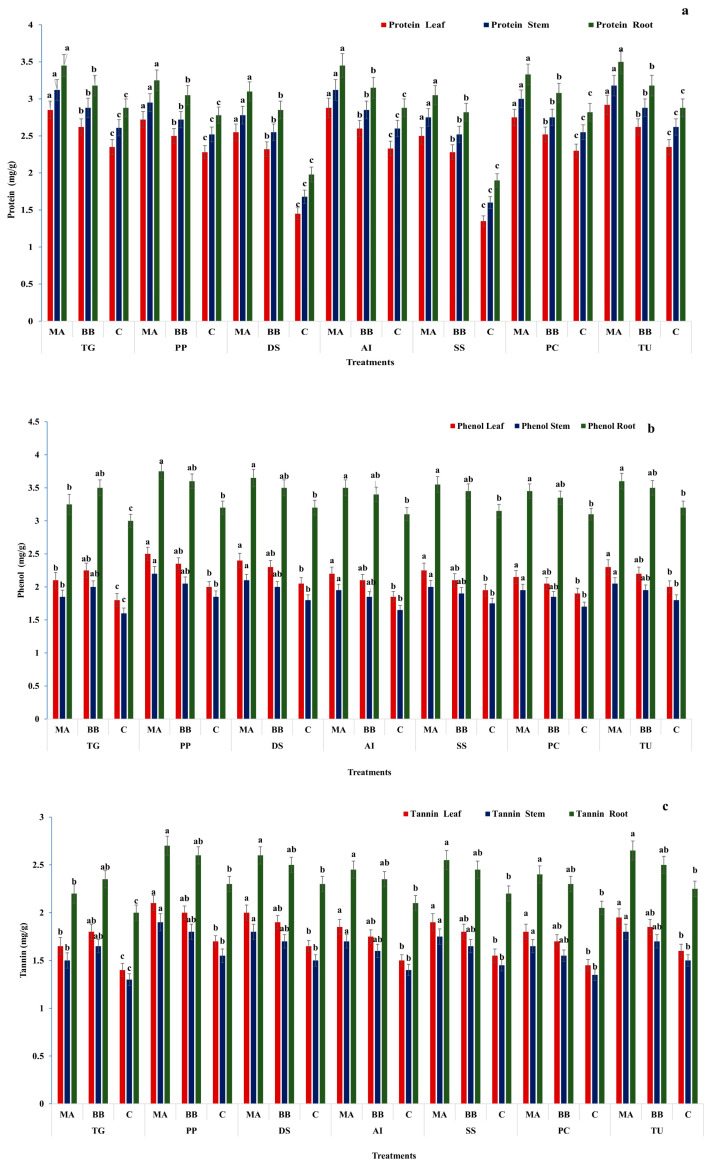
Biochemical responses of tree species [*Tectona grandis* (TG), *Pongamia pinnata* (PP), *Dalbergia sissoo* (DS), *Azadirachta indica* (AI), *Senegalia senegal* (SS), *Prosopis cineraria* (PC), and *Tecomella undulata* (TU)] to fungal inoculation. Protein (**a**), total phenol (**b**), and tannin (**c**) contents (mg g^−1^) were quantified in leaves, stems, and roots following treatment with *Metarhizium anisopliae*, *Beauveria bassiana*, and untreated control. The results indicate variations in biochemical constituents among plant parts and treatments, reflecting differential plant responses to fungal inoculation. Values are expressed as mean ± standard error (SE). Different lowercase letters (a–c) indicate statistically significant differences among treatments within each plant species and tissue at *p* ≤ 0.05.

Protein content was significantly higher in fungal-treated plants, particularly under *M. anisopliae*. In *T. grandis*, protein content increased to 2.9, 3.1, and 3.5 mg g^−1^ (leaves, stems, roots), which was significantly higher than *B. bassiana* (2.6, 2.9, and 3.2 mg g^−1^) and controls (2.4, 2.6, and 2.9 mg g^−1^). Similar trends were observed across *P. pinnata*, *D. sissoo*, *A. indica*, *S. senegal*, *P. cineraria*, and *T. undulata*, with *M. anisopliae* inducing significantly higher protein accumulation. The highest protein content was recorded in *T. undulata* (2.9, 3.2, and 3.5 mg g^−1^). Phenolic content was also significantly higher following fungal colonization. In *P. pinnata*, phenol levels under *M. anisopliae* reached 2.5, 2.2, and 3.8 mg g^−1^, significantly higher than controls and comparable to or higher than *B. bassiana*. Similar significant increases were observed in *D. sissoo* and *A. indica*. However, in certain cases (e.g., roots of *T. grandis*), *B. bassiana* showed slightly higher phenol content than *M. anisopliae*, indicating tissue-specific variation. Nevertheless, both fungi resulted in significantly higher phenolic content than controls. Tannin content followed a similar trend, with fungal treatments resulting in significantly higher levels than controls across all species. *M. anisopliae* generally induced significantly higher tannin content, particularly in roots and leaves. In *A. indica*, tannin content reached 1.9, 1.7, and 2.5 mg g^−1^ under *M. anisopliae*, which was significantly higher than *B. bassiana* and controls. Similar significant increases were observed in *P. pinnata* and *T. undulata*. Protein, phenol, and tannin contents were significantly higher in roots compared to stems and leaves. Across all species and tissues, *M. anisopliae* consistently induced significantly higher biochemical responses than *B. bassiana*, indicating its stronger role in enhancing host defense metabolism following endophytic colonization (Supplementary Table S1) (Figure 1a–c).

### 2.5. Induction of Defense-Related Enzymes in Tree Seedlings Following Endophytic Fungal Inoculation

**Figure 2:**
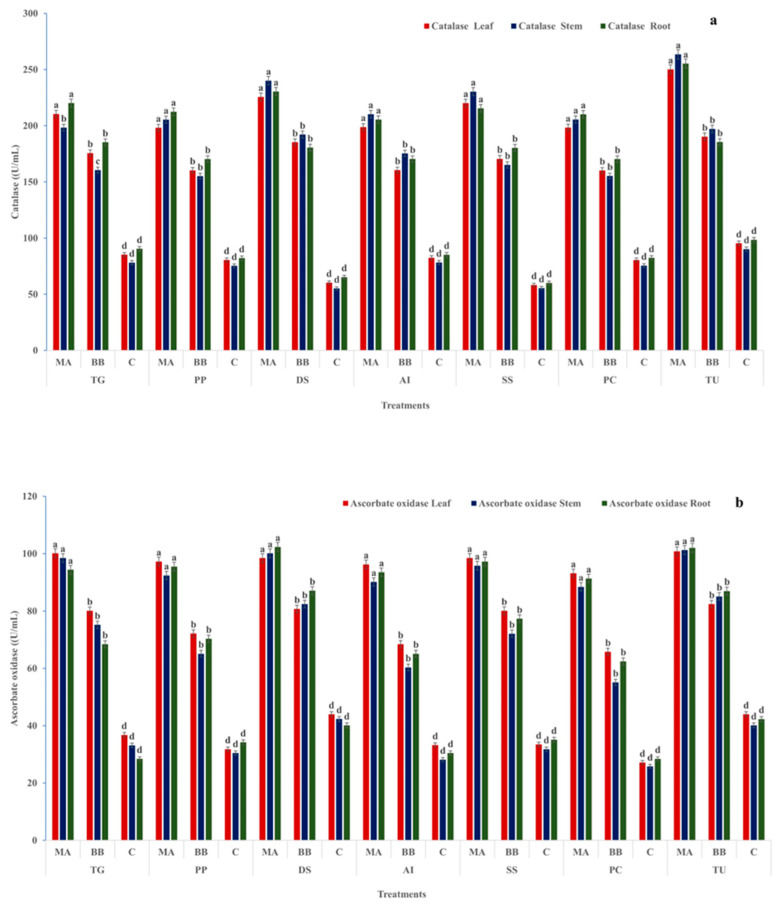
Enzymatic antioxidant responses of tree species [*Tectona grandis* (TG), *Pongamia pinnata* (PP), *Dalbergia sissoo* (DS), *Azadirachta indica* (AI), *Senegalia senegal* (SS), *Prosopis cineraria* (PC), and *Tecomella undulata* (TU)] under fungal treatments. Catalase activity (**a**) and ascorbate oxidase activity (**b**) were measured in leaves, stems, and roots of selected tree species following inoculation with *Metarhizium anisopliae* (MA), *Beauveria bassiana* (BB), and untreated control (C). The results demonstrate differential enzymatic antioxidant responses among plant parts and treatments, reflecting variation in induced defense mechanisms. Values are expressed as mean ± standard error (SE). Different lowercase letters (a–d) indicate statistically significant differences among treatments within each plant species and tissue at *p* ≤ 0.05.

**Figure 3:**
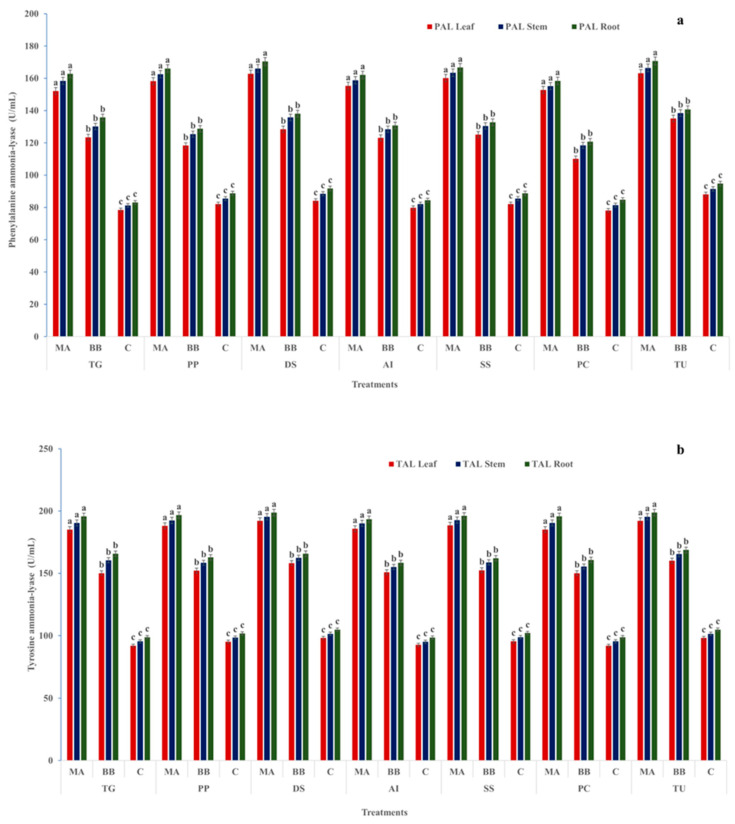
Defense-related enzyme activities of tree species [*Tectona grandis* (TG), *Pongamia pinnata* (PP), *Dalbergia sissoo* (DS), *Azadirachta indica* (AI), *Senegalia senegal* (SS), *Prosopis cineraria* (PC), and *Tecomella undulata* (TU)] under fungal treatments. Phenylalanine ammonia-lyase (PAL) activity (**a**) and tyrosine ammonia-lyase (TAL) activity (**b**) were measured in leaves, stems, and roots of the studied tree species following inoculation with *Metarhizium anisopliae* (MA), *Beauveria bassiana* (BB), and untreated control (C). The results highlight treatment-induced variation in key defense-related enzymes across plant parts. Values are expressed as mean ± standard error (SE). Different lowercase letters (a–c) indicate statistically significant differences among treatments within each plant species and tissue at *p* ≤ 0.05.

Catalase (CAT) activity was significantly higher in fungal-treated plants, particularly under *M*. *anisopliae*. In *T. grandis*, CAT activity reached 210.5, 198.3, and 220.2 U mL^−1^ (leaves, stems, roots), which was significantly higher than *B*. *bassiana* (175.7–185.3 U mL^−1^) and controls (78.1–90.5 U mL^−1^). Similar trends were observed in *P. pinnata* (198.1–212.5 U mL^−1^ vs. 155.1–170.4 and 75.3–82.2 U mL^−1^). In *D. sissoo* and *S. senegal*, CAT activity was also significantly higher under *M. anisopliae* (>215.0 U mL^−1^). The highest CAT activity was recorded in *T. undulata* (250.1, 263.5, and 255.3 U mL^−1^), significantly exceeding all other treatments. Ascorbate oxidase (AO) activity followed a similar pattern. In *T. grandis*, AO activity under *M. anisopliae* (94.4–100.1 U mL^−1^) was significantly higher than *B. bassiana* (68.4–80.1 U mL^−1^) and controls (28.5–36.8 U mL^−1^). In *D. sissoo*, AO activity reached 98.4–102.3 U mL^−1^ under *M. anisopliae*, significantly higher than *B. bassiana* (80.8–87.1 U mL^−1^) and controls (~40.0–44.0 U mL^−1^) (Figure 2a,b). *T. undulata* showed the highest AO activity. Phenylalanine ammonia-lyase (PAL) activity was significantly higher under *M. anisopliae*. In *P. pinnata*, PAL ranged from 158.2 to 166.1 U mL^−1^, compared with 118.3–128.8 U mL^−1^ under *B. bassiana* and 82.1–88.8 U mL^−1^ in controls. In *D. sissoo*, PAL activity (162.8–170.5 U mL^−1^) was significantly higher than *B. bassiana* (128.5–138.1 U mL^−1^). *T. undulata* exhibited the highest PAL activity (163.1–170.8 U mL^−1^). Tyrosine ammonia-lyase (TAL) activity was also significantly higher under *M. anisopliae*. In *A. indica*, TAL ranged from 185.8 to 193.5 U mL^−1^, significantly higher than *B. bassiana* (150.8–158.5 U mL^−1^) and controls (92.8–98.5 U mL^−1^). In *P. cineraria*, TAL (185.1–195.8 U mL^−1^) was significantly higher than *B. bassiana* (150.1–160.8 U mL^−1^), while *T. undulata* showed the highest activity (192.1–198.8 U mL^−1^) (Figure 3a,b). Enzyme activities were significantly higher in roots and stems than in leaves, particularly under soil-mediated colonization. Across all species and tissues, *M. anisopliae* induced significantly higher oxidative and phenylpropanoid enzyme activities than *B. bassiana*, indicating enhanced activation of host defense metabolism (Supplementary Table S2).

### 2.6. Termite Mortality and Mycosis Following Feeding on EPF-Treated Seedling Tissues

Worker termite mortality after 96 h of feeding on EPF-treated seedlings was higher in all fungal treatments compared to controls (6.5–9.2% in termites fed on root and stem tissues from non-inoculated seedlings). Across all tree species, *M. anisopliae* induced greater mortality in both root- and stem-fed termites than *B. bassiana*. The highest mortality was observed in *P. cineraria* (87.3% root; 90.2% stem) and *A. indica* (85.2% root; 88.7% stem) under *M. anisopliae* treatment. Stem-fed termites generally exhibited slightly higher mortality than root-fed termites. Mycosis followed a similar pattern to mortality. Termites fed on *M. anisopliae*-treated tissues showed higher fungal outgrowth, reaching up to 74.0% in stem-fed termites of *P. cineraria*, whereas *B. bassiana* produced comparatively lower mycosis (maximum 66.8%). No mycosis was recorded in the control treatment, where termites were fed with tissues from non-inoculated seedlings, confirming the absence of fungal infection. EPF-treated plants, particularly those inoculated with *M. anisopliae*, effectively induced termite mortality and confirmed fungal infection (Supplementary Table S3).

### 2.7. Coordinated Biochemical Defense Networks and Multivariate Patterns of Tissue-Specific Activation

**Figure 4:**
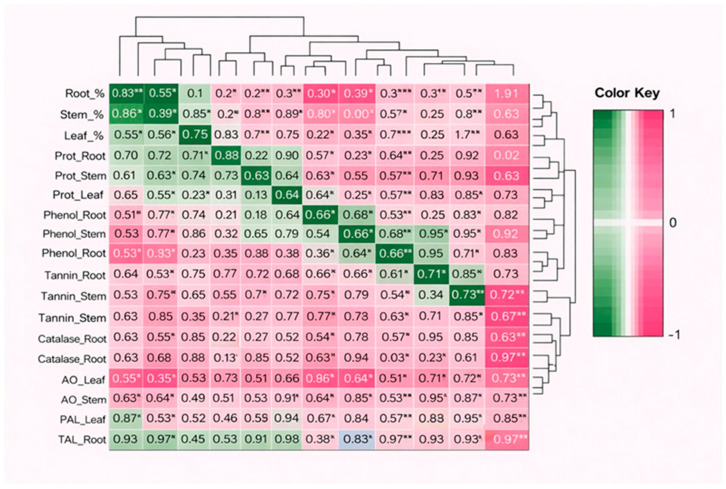
Hierarchical clustering heatmap showing Pearson’s correlation coefficients among biochemical defense parameters and tissue colonization percentages in EPF-treated seedlings. The color scale represents the strength and direction of correlation (green = positive correlation; pink/red = negative correlation), with values ranging from -1 to +1. Dendrograms indicate clustering patterns among variables based on similarity. Asterisks denote levels of statistical significance (* *p* < 0.05; ** *p* < 0.01; *** *p* < 0.001). Prot = total protein, AO = ascorbate oxidase, PAL = phenylalanine ammonia-lyase, and TAL = tyrosine ammonia-lyase.

Pearson’s correlation analysis revealed several strong and statistically significant positive associations among biochemical defense parameters across tissues. In root tissues, Catalase_Root showed a very strong positive correlation with AO_Root (*r* = 0.969, *p* < 0.001), PAL_Root (*r* = 0.952, *p* < 0.001), and TAL_Root (*r* = 0.961, *p* < 0.001). Root_% was significantly correlated with Catalase_Root (*r* = 0.943, *p* < 0.001), PAL_Root (*r* = 0.918, *p* < 0.001), and TAL_Root (*r* = 0.926, *p* < 0.001), indicating strong alignment between root damage response and antioxidant enzyme activity. Phenol_Root was significantly correlated with Tannin_Root (*r* = 0.889, *p* < 0.001). While in stem tissues, Catalase_Stem exhibited strong positive correlations with AO_Stem (*r* = 0.958, *p* < 0.001), PAL_Stem (*r* = 0.936, *p* < 0.001), and TAL_Stem (*r* = 0.944, *p* < 0.001). Stem_% was significantly associated with Catalase_Stem (*r* = 0.951, *p* < 0.001), PAL_Stem (*r* = 0.908, *p* < 0.001), and TAL_Stem (*r* = 0.919, *p* < 0.001). Phenol_Stem and Tannin_Stem were also significantly correlated (*r* = 0.864, *p* < 0.001). Further, in leaf tissues, Catalase_Leaf showed very strong correlations with AO_Leaf (*r* = 0.972, *p* < 0.001), PAL_Leaf (*r* = 0.948, *p* < 0.001), and TAL_Leaf (*r* = 0.955, *p* < 0.001). Leaf_% was significantly correlated with Catalase_Leaf (*r* = 0.947, *p* < 0.001), PAL_Leaf (*r* = 0.902, *p* < 0.001), and AO_Leaf (*r* = 0.931, *p* < 0.001). Phenol_Leaf was positively correlated with Tannin_Leaf (*r* = 0.871, *p* < 0.001). Across tissues, PAL and TAL activities showed consistently strong correlations (Root: *r* = 0.968; Stem: *r* = 0.962; Leaf: *r* = 0.965; all *p* < 0.001), confirming tight coordination of phenylpropanoid pathway enzymes. No strong significant negative correlations (|*r*| > 0.70) were observed, indicating synergistic rather than antagonistic regulation among defense traits (Figure 4).

### 2.8. Tissue-Specific Principal Component Analysis of Biochemical Defense Traits

**Figure 5:**
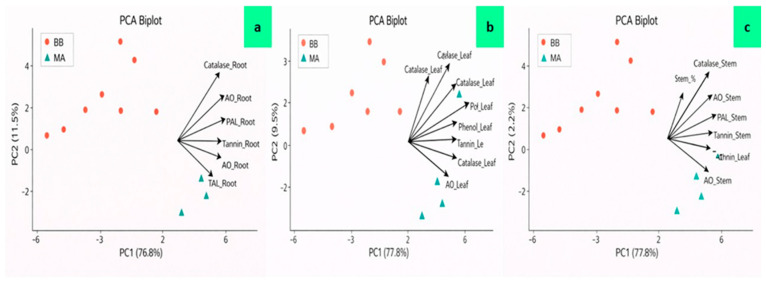
Principal Component Analysis (PCA) biplots illustrating tissue-specific biochemical defense responses in EPF-treated seedlings. (**a**) Root tissues, (**b**) leaf tissues, and (**c**) stem tissues. PC1 explains the majority of total variance (roots: 76.8%; leaves: 77.8%; stems: 77.8%), while PC2 accounts for secondary variation (roots: 11.5%; leaves: 9.5%; stems: 2.2%). Arrows represent variable loadings of enzymatic (catalase, AO, PAL, TAL) and phenolic defense traits, while points indicate treatment groups (MA: *M. anisopliae*; BB: *B. bassiana*). Separation along PC1 reflects coordinated biochemical defense activation under fungal inoculation. AO = ascorbate oxidase, PAL = phenylalanine ammonia-lyase, and TAL = tyrosine ammonia-lyase.

In roots, PC1 explained 76.8% of the total variance and represented a dominant biochemical defense activation axis. High positive loadings were observed for catalase, antioxidant activity (AO), PAL, TAL, phenols, and tannins, with Root_% closely aligned with these traits. MA-treated seedlings showed higher PC1 scores than BB controls, indicating stronger enzymatic and phenolic activation. PC2 accounted for 11.5% of the variance and was mainly associated with protein content and minor tannin variation, reflecting species-level metabolic differences. Together, PC1 and PC2 explained 88.3% of root variability. Further, in leaves, PC1 explained 77.8% of the total variance and clearly separated MA and BB treatments. Catalase, AO, PAL, TAL, phenols, and Leaf_% loaded strongly on PC1, confirming coordinated defense induction under MA treatment. PC2 explained 9.5% of the variance and was associated primarily with protein and minor tannin variation, indicating secondary metabolic adjustments. Combined, PC1 and PC2 accounted for 87.3% of total leaf variation. While, in stems, PC1 also explained 77.8% of total variance and was strongly influenced by catalase, PAL, TAL, AO, and phenolic content. Stem_% aligned with these defense-related traits, and MA-treated plants clustered at higher PC1 values. PC2 explained only 2.2% of the variance, indicating minimal secondary variation. Together, PC1 and PC2 accounted for 80.0% of stem variability, highlighting the dominance of treatment-driven enzymatic and phenolic activation in stem tissues (Figure 5).

### 2.9. Molecular Docking Interaction of Fungal Metabolites with Odontotermes cellulase

**Figure 6:**
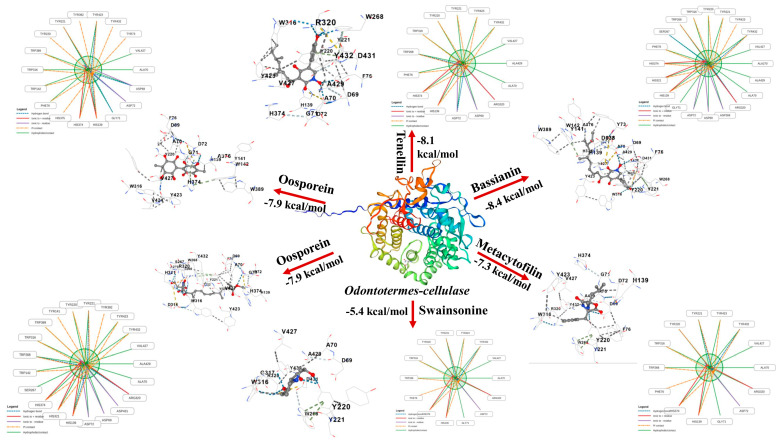
Molecular docking interactions between fungal secondary metabolites and cellulase of *Odontotermes*. The interactions between the ligand and the targeted protein are represented by colored dots as follows: the orange dot indicates a cation-π interaction; the green dot represents π-π stacking; the grey dot denotes hydrophobic contact; the yellow dot corresponds to ionic interactions; the blue dot indicates hydrogen bonds; and the light blue dot represents weak hydrogen bonds.

The predicted three-dimensional structure of the cellulase protein of *Odontotermes* generated through SWISS-MODEL was evaluated for structural reliability before performing docking analysis. Validation using the Ramachandran plot through PROCHECK available on the SAVES v6.0 server indicated that 94.62% of amino acid residues were located in the most favored regions, confirming the good stereochemical quality and reliability of the predicted protein model for subsequent docking studies. Molecular docking between the selected fungal secondary metabolites and the cellulase protein was performed using the CB-Dock2 server, which utilizes the AutoDock Vina 1.2.0 algorithm for blind docking and cavity detection. The docking results revealed that the selected metabolites exhibited varying binding affinities toward the cellulase active site (Figure 6). Among the tested compounds, Bassianin showed the highest binding affinity with a binding energy of −8.4 kcal/mol, indicating a strong interaction with the cellulase protein. This was followed by Tenellin with a docking score of −8.1 kcal/mol. Both compounds formed multiple stabilizing interactions with key amino acid residues within the binding pocket of the target protein. Oosporein displayed binding affinities of −7.9 kcal/mol, suggesting moderate but stable binding interactions with the cellulase enzyme. Another compound, Metacytofilin, demonstrated a binding energy of −7.3 kcal/mol, indicating relatively weaker but still significant interaction with the target protein. In contrast, Swainsonine exhibited the lowest binding affinity among the selected metabolites with a docking score of −5.4 kcal/mol, suggesting comparatively weaker interaction with the cellulase active site. Interaction analysis revealed that these ligands formed several hydrogen bonds, hydrophobic interactions, and π-interactions with key amino acid residues such as His139, Asp69, Tyr221, Tyr423, Trp316, Val427, and Ala429, which are located within the predicted binding cavity of the cellulase protein. These interactions contribute to the stabilization of ligand–protein complexes and may influence the inhibition of cellulase activity.

## 3. Discussion

The present study demonstrates that EEPF functions as a powerful and multifunctional strategy for the management of *O. obesus* through both direct pathogenicity and indirect enhancement of host plant defense mechanisms. By integrating virulence bioassays, colonization assessments, biochemical profiling, and defense enzyme analyses, the findings clearly establish that fungal–plant symbiosis significantly suppresses termite pressure while strengthening intrinsic plant resistance. These results are consistent with earlier studies showing that EPF contribute not only to insect mortality but also to enhanced plant resilience and ecosystem sustainability [23,24,25,26]. Among the two fungi tested, *M. anisopliae* exhibited significantly greater virulence against *O. obesus* than *B. bassiana*. The shorter LT_50_ and LT_90_ values recorded for *M. anisopliae* indicate rapid termite mortality, which is particularly critical when targeting eusocial insects whose colony survival depends on coordinated worker function. Rapid mortality disrupts foraging efficiency, brood care, and structural maintenance within the colony. The steeper probit slope and lower heterogeneity factor further indicate greater uniformity in host susceptibility and consistent pathogenic action. Narrow fiducial limits validate the reliability of these estimates. The superior pathogenicity of *M. anisopliae* may be attributed to enhanced spore adhesion, stronger cuticle-degrading enzyme activity, and rapid internal colonization of termite tissues [3,4,9]. These observations align with earlier findings, where Kamarudin et al. (2022) [26] reported rapid mortality of *Coptotermes curvignathus* following exposure to *M. anisopliae*. Singha et al. (2011) [27] demonstrated lower LC_50_ values for *M. anisopliae* compared with *B. bassiana* against *Microtermes obesi*, and Ambele et al. (2020) [28] identified *Metarhizium* isolates as highly virulent against subterranean termites. The present results therefore reinforce the suitability of *M. anisopliae* as a core biocontrol agent for termite management.

A key outcome of this investigation is the demonstration that both fungi can establish as endophytes in multiple forest tree species, with *M. anisopliae* consistently achieving higher colonization frequencies than *B. bassiana*. Soil application resulted in more effective and uniform colonization than foliar spray, particularly in roots. This pattern can be explained by the intimate interaction between fungal propagules and the rhizosphere environment, where root exudates facilitate spore germination and tissue penetration [2,5,6,9]. Once established, *M. anisopliae* appears capable of persistent root colonization with limited but measurable systemic movement. The stronger root colonization observed across species suggests ecological adaptation of *M. anisopliae* to soil habitats and woody perennials. Similar host- and method-dependent colonization patterns have been documented by Ambele et al. (2020) [28], Biswas et al. (2013) [29], and Greenfield et al. (2016) [30]. The consistently higher colonization of *M. anisopliae* indicates greater compatibility with forest tree systems, particularly in nursery conditions where root protection is essential [2].

Biochemical analyses revealed that EEPF inoculation significantly enhanced total protein, phenol, and tannin content across tissues and species, with the most pronounced increases observed in roots. The higher accumulation of secondary metabolites in roots is ecologically meaningful, given that subterranean termites initiate feeding below ground and directly attack root systems [31]. Enhanced root phenol and tannin levels are likely to reduce palatability and interfere with termite digestive enzymes at the primary site of attack [32]. Tannins can bind to dietary proteins in the termite gut, limiting nutrient assimilation and weakening colony growth [33]. Increased protein content in roots may reflect elevated synthesis of defense-related proteins and stress-responsive enzymes associated with induced resistance. The strong species × EPF interactions observed in the analysis of variance suggest that host genotype influences biochemical responsiveness, with species such as *T. undulata*, *D. sissoo*, and *A. indica* showing particularly strong induction. These findings are consistent with earlier reports indicating that EPF stimulate the synthesis of defense-related secondary metabolites [34,35,36].

Defense enzyme activity patterns further support the central role of roots in induced resistance. Catalase, ascorbate oxidase, phenylalanine ammonia-lyase, and tyrosine ammonia-lyase were significantly elevated following fungal inoculation, with the highest activities recorded in root tissues. This root-centered enzyme activation suggests localized defense intensification at the primary interface between plant and termite attack [37]. CAT and AO regulate reactive oxygen species generated during biotic stress, thereby maintaining redox balance and preventing oxidative damage in actively colonized tissues [38]. The elevated antioxidant activity in roots likely reflects both fungal colonization and termite-associated stress signals. PAL and TAL, key regulatory enzymes of the phenylpropanoid pathway, initiate the synthesis of phenolics, lignin precursors, and tannins [36,37,38]. Their strong induction in roots explains the higher accumulation of phenolic and tannin compounds observed in these tissues. Enhanced lignification and phenolic deposition in roots may strengthen cell walls, making penetration and feeding more difficult for termites [39]. Comparable activation of antioxidant and phenylpropanoid enzymes in EPF-colonized plants has been reported by Tomilova et al. (2020) [40] and Vivekanandhan et al. (2025) [41], highlighting the importance of enzyme-mediated defense modulation.

Furthermore, the present study confirms that endophytic entomopathogenic fungi (*M. anisopliae* and *B. bassiana*) not only act as direct biocontrol agents against the subterranean termite *O. obesus* but also induce coordinated biochemical defense responses in host seedlings, supporting their multifunctional potential in sustainable pest management. Endophytic colonization by EPF is a recognized mechanism through which fungi confer protection against herbivores and enhance plant resilience, consistent with documented roles of EPF in protecting plants from insect pests and stressors [42]. In line with recent findings, *M. anisopliae* exhibited stronger virulence than *B. bassiana*, leading to higher termite mortality and greater mycosis in feeding bioassays [43]. Comparable patterns of enhanced mortality from entomopathogenic fungi (*Fusarium solani and F. falciforme*) exposure have been reported in *Coptotermes* spp. and other soil-dwelling pests [44]. The absence of infection in controls further validates the necessity of fungal presence for mortality, underscoring the dual role of EPF colonization in both direct pathogenicity and indirect insect suppression [45]. Beyond pathogenic effects, our biochemical analyses reveal that EPF colonization induces significant upregulation of antioxidant enzymes (catalase, AO) and key phenylpropanoid pathway enzymes (PAL, TAL) across root, stem, and leaf tissues. Enhanced phenolic and tannin contents following EPF inoculation mirror recent studies showing that endophytic associations stimulate host antioxidant and secondary metabolite responses, thereby strengthening defense against biotic stressors [2,3,4,5,6]. The strong positive correlations among defense enzymes and colonization levels observed here suggest synergistic activation of systemic resistance rather than isolated metabolic shifts [42,43,44,45,46].

Principal component analyses further corroborate this coordinated defense model, with the dominant axes driven by enzyme activities and phenolic accumulation, clearly segregating *M. anisopliae*-treated plants from *B. bassiana* and controls. Such multivariate patterns of biochemical induction are indicative of effective systemic defense enhancement and align with broader concepts of endophyte-mediated resistance wherein endophytic microbes act as elicitors of plant defensive networks [46]. Collectively, these findings extend the understanding of EPF beyond classical insect pathology by demonstrating their contribution to induced plant defense activation. By combining virulence and defense priming, EPF-like *M. anisopliae* represent a promising dual-function biocontrol strategy for protecting forest seedlings against termite damage while enhancing host physiological robustness. Future work may focus on field validation and optimization of inoculation techniques to translate these synergistic mechanisms into scalable pest management solutions. Furthermore, the docking results indicate that fungal secondary metabolites possess strong potential to interfere with termite digestive physiology. Among the evaluated compounds, Bassianin exhibited the highest binding affinity toward the cellulase enzyme (−8.4 kcal/mol), followed by Tenellin (−8.1 kcal/mol) and Oosporein (−7.9 kcal/mol), suggesting stable ligand–protein complexes. The formation of hydrogen bonds and hydrophobic interactions with key residues such as His139, Asp69, Tyr221, Tyr423, Trp316, Val427, and Ala429 within the binding cavity indicates that these metabolites may effectively block or alter the catalytic activity of cellulase. Since cellulase enzymes play a crucial role in lignocellulose degradation and nutrient acquisition in termites, disruption of this enzymatic system could significantly impair feeding efficiency and colony survival. Previous studies have shown that entomopathogenic fungi produce diverse bioactive metabolites capable of targeting essential metabolic pathways in insects [47]. In termites, enzymes involved in digestion and detoxification, including cellulases and cytochrome P450 systems, are critical for survival and adaptation [48,49,50]. Therefore, targeting these proteins represents a promising strategy for termite management. In contrast to conventional synthetic termiticides, which often cause environmental contamination, bioaccumulation, and adverse effects on non-target organisms [51], fungal metabolites provide an environmentally safer alternative. Similar in silico approaches have also been successfully applied to identify potential insecticidal compounds targeting insect proteins.

## 4. Materials and Methods

### 4.1. Collection and Maintenance of Termites

Worker termites (*Odontotermes obesus*) were collected from infested wood debris and termite mounds in plantation areas of Khejri and Neem at the ICFRE–Arid Forest Research Institute, Jodhpur, Rajasthan, India (26.228744° N, 73.031412° E) during the 2023–2024 sampling period (March–April). The collected termites were transported to the laboratory in ventilated plastic containers containing field-collected wood and soil to minimize handling stress. In the laboratory, termites were maintained in plastic containers containing sterilized soil and moistened filter paper. The containers were kept in an incubator at 26 ± 2 °C and 75 ± 5% relative humidity under dark conditions. Prior to bioassays, termites were acclimatized for 1 h, and only active, healthy, and uninjured worker termites of uniform size were selected to ensure a standardized test population.

### 4.2. Preparation of Fungal Cultures and Conidial Suspensions

Stock cultures of *M. anisopliae* NBAIR-MaCB (Accession number: MN727141.1) and *B. bassiana* (*sensu lato*) were obtained from the ICAR–National Bureau of Agricultural Insect Resources (NBAIR), Bengaluru, Karnataka, India. *M. anisopliae* was cultured on Sabouraud Dextrose Agar (SDA) (HiMedia, Mumbai, India), while *B. bassiana* was cultured on Potato Dextrose Agar (PDA) (HiMedia, Mumbai, India) in 90 mm Petri dishes. Cultures were incubated at 25 ± 1 °C in complete darkness for 2–3 weeks to allow adequate sporulation. Conidia were harvested by gently scraping the colony surface using a sterile spatula and suspended in 10 mL sterile distilled water containing 0.05% Triton X-100 (Sigma-Aldrich, St. Louis, MO, USA). The suspension was vortexed for 5 min to ensure uniform dispersion and filtered through sterile cheesecloth to remove mycelial debris. The filtrate was centrifuged at 55× *g* (∼700 rpm, 10 cm rotor radius) for 5 min. The resulting conidial pellet was then resuspended in sterile distilled water. Conidial concentration was determined using a Neubauer haemocytometer (Marienfeld, Lauda-Königshofen, Germany) and adjusted to 1 × 10^8^ conidia mL^−1^ for bioassays. For each fungal isolate, four replicate culture plates were used. Conidial viability was assessed prior to application using a germination test, ensuring >90% viability for experimental use.

### 4.3. Evaluation of Fungal Isolates Against Termites

The pathogenicity of fungal isolates was tested on worker termites of *O. obesus*. Each fungal isolate was evaluated in triplicate along with a control group. Twenty worker termites were placed in round plastic containers (9 cm diameter × 5 cm height). To maintain normal social interaction and behavioral cohesion during the acclimatization phase, two soldier termites were initially included in each container; however, these were carefully removed prior to the application of treatments and initiation of the bioassay. The worker termites were then sprayed with 1 mL of a 1 × 10^8^ conidia mL^−1^ suspension using a Potter spray tower (MRP-PT-M spray, IndiaMART, New Delhi, India). Control groups were treated with sterile distilled water containing 0.05% Triton X-100. All termite groups (both fungal treatments and control) were provided with sterilized wood derived from the same plant species used in this experiment, along with soil substrate for food and shelter under identical conditions. No alternative or different food source was used for the control group. The containers were incubated at 23 ± 2 °C and 75 ± 5% relative humidity under dark conditions. Mortality was recorded at 24 h post-treatment and subsequently at 12 h intervals (36, 48, 60, and 72 h). Observations were continued up to 72 h, and the experiment was replicated four times. The pathogenic effects were assessed based solely on worker termite mortality following treatment with *B. bassiana* and *M. anisopliae*.

### 4.4. LT_50_ Calculation and Confirmation of Fungal Infection

The LT_50_ (time required to achieve 50% mortality) values were calculated using Abbott’s formula (1925) [52]. To verify that fungal infection caused termite mortality, dead termites were surface sterilized and placed on sterile Whatman filter paper (Cytiva, Marlborough, MA, USA) in Petri dishes. The presence of fungal infection was confirmed through daily microscopic examination of hyphal and spore development.

### 4.5. Nursing of Selected Forest Seedlings

Seeds of *Dalbergia sissoo* Roxb., *Azadirachta indica* A. Juss., *Prosopis cineraria* (L.) Druce, *Senegalia senegal* (L.) Britton, *Tectona grandis L.f.*, *Tecomella undulata* (Sm.) Seem., and *Pongamia pinnata* (L.) Pierre. were obtained from the nursery of the ICFRE—Arid Forest Research Institute, Jodhpur, Rajasthan, for inoculation studies during 2023–2024. Seeds were surface sterilized by immersing in 70% ethanol, followed by treatment with 1.5% sodium hypochlorite solution for 2–3 min. After disinfection, the seeds were rinsed multiple times to remove residual chemicals. The sterilized seeds were then air-dried under laminar air flow to prevent contamination. Subsequently, seeds were sown in sterilized pots containing growth medium composed of soil, sand, and farmyard manure mixed in equal proportions (1:1:1), and one seedling per pot was maintained for uniform growth conditions.

### 4.6. Seedling Inoculation Procedures

Fifteen days after germination, at the two-leaf stage, the seedlings were inoculated using two methods: foliar spraying and soil drenching. For the soil drench method, each seedling’s root zone received 25 mL of the prepared conidial suspension, while control seedlings received an equivalent treatment with sterile water containing 0.05% Triton X-100. For foliar spraying, each seedling’s leaves were sprayed with 25 mL of the conidial suspension, and control seedlings were sprayed with water only. After inoculation, seedlings were maintained in a screened house under ICFRE-AFRI nursery conditions and provided with two weekly irrigations. Each fungal isolate was replicated four times, and the treatments were arranged in a completely Randomized Design (CRD).

### 4.7. Assessment of Endophytic Colonization

Colonization rates were evaluated two months after inoculation. For this assessment, three independent plants (biological replicates) per fungal isolate were carefully uprooted and thoroughly rinsed under running water to remove any adhering soil. Leaf samples were collected for the foliar inoculation method, and root samples for the soil incorporation method. These samples were cut into small segments (approximately 1.0–1.5 cm), surface-sterilized, and placed onto SDA or PDA plates supplemented with antibiotics (streptomycin sulfate, 100 µg/mL) (HiMedia, Mumbai, India) to prevent bacterial growth. The plates were incubated at 25 °C and observed daily for fungal growth over a period of 25 days. Each fungal isolate was evaluated in four independent experimental replicates, and multiple tissue segments from each plant were plated, providing a large sample size for colonization assessment. Colonization was quantified by recording the number of tissue segments showing fungal growth, and the percentage colonization was calculated following the method of Fisher and Petrini (1987) [53]: Colonization % = (Number of pieces showing fungal growth/Total pieces plated) × 100.

### 4.8. Plant Sample Collection

Two months after inoculation, three leaves, stems, and root samples (from the soil incorporation method) were collected from both inoculated and non-inoculated (control) seedlings of each tree species (*D. sissoo*, *S. senegal*, *A. indica*, *P. cineraria*, *T. grandis*, *T. undulata*, and *P. pinnata*). For each treatment, fully expanded and healthy leaves, along with fine lateral roots and stem tissues from the mid-region of the plants, were selected to ensure uniformity and minimize variability due to tissue age and position. The collected samples were gently washed with distilled water to remove adhering soil particles and surface contaminants. The tissues were then blot-dried with sterile paper towels, placed in clean, properly labeled polythene bags, and immediately transported to the laboratory under cooled conditions to prevent the degradation of sensitive metabolites and enzymatic activities. Upon arrival, the fresh samples were stored at 4 °C until further biochemical and enzymatic analyses.

### 4.9. Sample Preparation for Biochemical Analysis

Leaf, stem, and root tissues were collected separately from each treatment and analyzed in three independent replications following a completely randomized design. The fresh plant tissues were thoroughly washed with distilled water, blotted dry, and finely ground in liquid nitrogen using a chilled mortar and pestle. The powdered samples were then homogenized in 10 mL of phosphate buffer (50 mM, pH 7.8) and centrifuged at 12,000 rpm for 20 min at 4 °C. The resulting clear supernatant was carefully collected and stored in 2.5 mL Eppendorf tubes (Eppendorf, Hamburg, Germany) at −20 °C until further biochemical analysis.

### 4.10. Estimation of Protein, Phenols and Tannins in Tree Seedlings

Total protein content was estimated using the Bradford (1976) [54] method, total phenols were determined by the Folin–Ciocalteu method as described by Singleton and Rossi (1965) [55], and tannin content was quantified following the method of Amorim et al., (2008) [56]. Absorbance for each biochemical parameter was measured using a UV–visible spectrophotometer, and concentrations were calculated from standard calibration curves prepared using bovine serum albumin for protein, gallic acid for phenols, and catechin for tannins. The results were expressed as mg g^−1^ fresh weight and used to evaluate the effect of endophytic colonization by entomopathogenic fungi on the biochemical profile of tree seedlings.

### 4.11. Estimation of Plant Defense Enzyme Activities in Tree Seedlings

Catalase (CAT) activity was determined following the method of Aebi (1984) [57] by monitoring the decrease in absorbance of hydrogen peroxide (H_2_O_2_) at 240 nm using a UV–visible spectrophotometer. The reaction mixture consisted of phosphate buffer, enzyme extract, and H_2_O_2_, and CAT activity was expressed as units per mL. Ascorbate oxidase (AO) activity was assayed according to Diallinas et al. (1997) [58] by measuring the oxidation of ascorbic acid at 265 nm, and the rate of decrease in absorbance was used to calculate enzyme activity, which was expressed as U mL^−1^. Phenylalanine ammonia-lyase (PAL) activity was determined using the method of Fritz et al. (1976) [59] with L-phenylalanine as substrate, and the formation of trans-cinnamic acid was measured at 290 nm; PAL activity was expressed as µmol trans-cinnamic acid formed per minute per mL. Tyrosine ammonia-lyase (TAL) activity was estimated following Beaun-Eagan and Thorpe (1985) [60] by measuring the formation of p-coumaric acid from L-tyrosine at 333 nm, and the activity was expressed as U mg^−1^ protein. These enzymatic parameters were used to assess the biochemical defense responses induced by endophytic colonization of entomopathogenic fungi in forest tree seedlings.

### 4.12. Termite Mortality and Mycosis After Feeding on EPF-Treated Plants

Worker termites of *O. obesus* were fed with root and stem tissues from seedlings previously inoculated with *M. anisopliae* and *B. bassiana* under standardized endophytic colonization procedures. After confirming fungal colonization, fresh root and stem tissues were excised, surface sterilized, and cut into uniform pieces (1.5 cm). In the control treatment, termites were provided with root and stem tissues derived from non-inoculated seedlings of the same plant species, which were processed identically (surface sterilized, cut into uniform pieces, and handled under the same sterile conditions). Groups of healthy worker termites (*n* = 30 per replicate) were placed in sterile Petri dishes lined with moistened filter paper and supplied with the respective plant tissues. Each treatment was conducted in triplicate under controlled laboratory conditions (27 ± 2 °C, 75 ± 5% relative humidity, and darkness). Mortality was recorded 96 h after feeding exposure. To confirm fungal infection, termite cadavers were surface sterilized using 0.1% sodium hypochlorite, rinsed thoroughly with sterile distilled water, and incubated on moist sterile filter paper in Petri dishes. Post-mortem mycosis was verified by observing characteristic hyphal growth and conidial sporulation emerging from the cadavers within 5–7 days. Percent mortality and percent mycosis were calculated based on the total number of exposed termites.

### 4.13. In Silico Molecular Docking of Entomopathogenic Fungal Metabolites with Termite Cellulase Protein

Secondary metabolites identified from *Metarhizium* spp. and *Beauveria bassiana* that satisfied Tice’s rule of insecticide-likeness were selected for molecular docking analysis [61]. The three-dimensional (3D) structures of the selected ligand molecules were retrieved from the PubChem database in Structure Data File (SDF) format (https://pubchem.ncbi.nlm.nih.gov/, accessed on 10 July 2025) [62]. Since the three-dimensional structure of cellulase protein of *Odontotermes* was not available in the Protein Data Bank (https://www.rcsb.org/, accessed on 10 July 2025) [63], the amino acid sequence of the target protein was obtained from the UniProt protein database (UniProt ID—D3JXK4) [64] (https://www.uniprot.org/, accessed on 15 September 2025). The retrieved protein sequence was used to generate a predicted 3D structure using the SWISS-MODEL server (https://swissmodel.expasy.org/, accessed on 15 September 2025) for homology modeling [65]. The quality and stereochemical properties of the generated protein model were validated using the Ramachandran plot through the SWISS-MODEL validation tool (https://swissmodel.expasy.org; accessed on 21 October 2025) and the PROCHECK module available in the SAVES v6.0 server [66]. The validated protein structure was downloaded in PDB format for subsequent molecular docking analysis. Molecular docking was performed using the CB-Dock2 server (https://cadd.labshare.cn/cb-dock2/, accessed on 15 September 2025). CB-Dock2 performs blind docking by integrating automatic cavity detection, docking, and homologous template fitting. The server identifies potential binding cavities within the target protein and predicts their center coordinates and dimensions. Docking calculations were carried out using the latest version of AutoDock Vina integrated within the CB-Dock2 platform. Ligand molecules (mol2, mol, sdf, or pdb format) and the target protein structure (pdb format) were uploaded to the server. The system automatically detected the binding cavities in the target protein and docked the ligands into the predicted active sites. The docking results were ranked based on Vina binding affinity scores, arranged from lowest to highest values. CB-Dock2 also provides three-dimensional visualization of ligand–protein interactions and docking conformations. The server has demonstrated docking accuracy with over 70% similarity to experimentally determined binding sites and an RMSD within 2 Å compared with X-ray crystallography data. The best docking conformations were selected based on the lowest binding energy and stable interaction patterns between the ligand and the target cellulase protein.

### 4.14. Statistical Analysis

Mortality data from termite bioassays were corrected for natural mortality in the control group using Abbott’s formula. For fungal isolates causing more than 50% mortality, time–mortality relationships were analyzed using a Generalized Linear Model with a binomial distribution and logit link function to estimate median lethal time (LT_50_) and 90% lethal time (LT_90_), along with their 95% confidence intervals (CIs). Probit regression analysis was additionally performed using PoloPlus software (version 2.0, LeOra Software, Berkeley, CA, USA) to support precise estimation of lethal time parameters. The incidence of mycosis and fungal colonization success was analyzed using one-way analysis of variance (ANOVA). Percentage data were subjected to arcsine square root transformation prior to analysis to stabilize variance, following Fisher and Petrini (1987) [53]. The experimental design followed a Completely Randomized Design (CRD) with at least three biological replicates per treatment. Biochemical and enzymatic parameters, including protein, phenols, tannins, and enzyme activities (catalase, ascorbate oxidase, phenylalanine ammonia-lyase, and tyrosine ammonia-lyase), were analyzed using a factorial CRD with three biological replicates. The experimental factors included seedling species (A), fungal inoculation (B: inoculated and non-inoculated control), and plant part (C: root, stem, and leaf). Accordingly, sources of variation included the main effects of A, B, and C, their two-way interactions (A × B, A × C, B × C), and the three-way interaction (A × B × C).

Prior to analysis, assumptions of normality and homogeneity of variance were evaluated using the Shapiro–Wilk test and Bartlett’s test, respectively. A three-way ANOVA was conducted to assess the effects of experimental factors on biochemical traits. When significant F-values were observed, treatment means were separated using Fisher’s Least Significant Difference test at *p* < 0.05. Relationships between fungal colonization and biochemical responses were examined using Pearson’s correlation analysis between tissue-specific colonization percentages (leaf, stem, and root) and corresponding biochemical parameters. Multiple linear regression analysis was performed with colonization percentage as the dependent variable and biochemical traits as predictors, and stepwise regression was used to identify the most influential variables contributing to colonization success and termite resistance. All statistical analyses were conducted using R software (version 4.4.1; R Core Team, Vienna, Austria). Prior to multivariate analysis, data were standardized using z-score transformation to ensure comparability among variables measured on different scales. Principal Component Analysis (PCA) was performed using the prcomp() function with scaling enabled to explore multivariate relationships among species, treatments, and plant parts. Eigenvalues, variance explained, and factor loadings were used to interpret principal components based on the Kaiser criterion (eigenvalue > 1) and scree plot analysis. Pairwise relationships among variables were further assessed using Pearson’s correlation coefficients, calculated with the rcorr() function from the Hmisc package, version 5.1-1. Statistical significance was determined using two-tailed tests (* *p* < 0.05, ** *p* < 0.01, *** *p* < 0.001), and correlation patterns were visualized using clustered heatmaps with hierarchical clustering (Euclidean distance, complete linkage). Path analysis was performed using Structural Equation Modeling with the lavaan package (version 0.6-17) to evaluate direct and indirect effects of biochemical traits on damage-related parameters. PCA-based dimensionality reduction was applied prior to modeling to avoid over-parameterization. Model fit was assessed using Comparative Fit Index, Root Mean Square Error of Approximation, and Standardized Root Mean Square Residual. For termite feeding assays, mortality and mycosis data were expressed as mean ± standard error (SE). Percentage data were arcsine square root transformed and analyzed using one-way ANOVA, with significance determined at *p* < 0.05.

## 5. Conclusions

The present study demonstrates the dual role of *M. anisopliae* and *B. bassiana* as effective endophytic entomopathogens for the sustainable management of *O. obesus* in forest tree seedlings. Among the tested fungi, *M. anisopliae* exhibited significantly higher virulence, faster lethal action, greater colonization efficiency, and more consistent termite mortality than *B. bassiana*. Soil inoculation significantly enhanced systemic colonization compared with foliar application. Endophytic establishment resulted in significantly higher protein, phenolic, and tannin contents, along with enhanced activities of defense-related enzymes, including catalase, phenylalanine ammonia lyase, tyrosine ammonia lyase, and ascorbate oxidase, indicating coordinated activation of antioxidant and phenylpropanoid pathways. Positive correlations between colonization levels and biochemical responses further support a mechanistic link between fungal establishment and host defense priming. Termite feeding assays confirmed increased mortality and mycosis in treated seedlings, while molecular docking suggested strong interactions of fungal metabolites (bassianin and tenellin) with termite cellulase, providing insights into possible modes of action. However, the study has certain limitations. Long-term persistence, stability of endophytic colonization, and ecological impacts on non-target soil and rhizosphere microbiota were not assessed. Additionally, molecular validation of defense pathways (gene expression or transcriptomic analyses) was not performed, and the docking results remain predictive without biochemical confirmation. Variability across environmental conditions, host genotypes, and large-scale applicability also requires further evaluation. Future research should therefore focus on transcriptomic and metabolomic validation, formulation optimization for field delivery, assessment of rhizosphere and non-target effects, and multi-location long-term field trials to establish the practical feasibility of these endophytic fungi as eco-friendly tools for integrated termite management in forestry systems.

## Figures and Tables

**Table 1:** Time-dependent mortality of *Odontotermes obesus* workers exposed to *Beauveria bassiana* and *Metarhizium anisopliae* under laboratory conditions.

Time	Total Insects	Total Mortality	Time	Total Insects	Total Mortality
*Beauveria bassiana*			*Metarhizium anisopliae*		
**C**	200	9	**C**	200	5
24 h	200	38	24 h	200	53
36 h	200	82	36 h	200	113
48 h	200	112	48 h	200	156
60 h	200	131	60 h	200	180
72 h	200	170	72 h	200	192
**Total (*n*)**	**1000**	**542**	**Total (*n*)**	**1000**	**699**

C = mortality in control after 72 h; total insects = number of termites used per observation (*n* = 200 per time interval; cumulative *n* = 1000); mortality values represent the number of dead termites recorded at each time point; observations were made at 24 h and subsequently at 12 h intervals up to 72 h under controlled laboratory conditions (23 ± 2 °C and 75 ± 5% RH).

**Table 2:** Median lethal time (LT_50_) and (LT_90_) of entomopathogenic fungi (EPFs) against *Odontotermes obesus*.

EPF	*n*	χ^2^ (df = 3)	*p*-Value	Slope ± SE	h	LT_50_ (95% FL) (h)	LT_90_ (95% FL) (h)	FL Width (h)	R^2^	Virulence Rank
*Metarhizium anisopliae*	1000	1.42	0.7	5.38 ± 0.27	0.46	33.12 (31.75–34.62)	60.84 (56.73–65.91)	29.09	0.95	1
*Beauveria bassiana*	1000	7.35	0.1	4.01 ± 0.25	2.31	46.78 (41.92–51.80)	101.42 (80.35–148.26)	59.5	0.93	2

*n* represents the total number of termites used in the bioassay for each entomopathogenic fungus (EPF). χ^2^ (df = 3) indicates the chi-square value of the probit regression goodness-of-fit test with three degrees of freedom. The *p*-value corresponds to the probability level associated with the chi-square statistic; *p* > 0.05 indicates an adequate model fit. Slope ± SE represents the slope of the probit regression line with its standard error, reflecting the rate and uniformity of mortality response. The heterogeneity factor (h) indicates the dispersion of observed responses relative to the model expectation; values close to 1 suggest good fit and low variability. LT_50_ and LT_90_ denote the median lethal time required to kill 50% and 90% of the termite population, respectively, expressed in hours (h). Values in parentheses represent 95% fiducial limits (FL). FL Width refers to the difference between upper and lower fiducial limits of LT_90_, indicating precision of estimation. R^2^ represents the coefficient of determination of the probit model. Virulence rank is assigned based on LT_50_ values, with lower LT_50_ indicating higher virulence.

## Data Availability

The raw data supporting the conclusions of this article will be made available by the authors on request.

## Supplementary Materials

The following supporting information can be downloaded at https://www.mdpi.com/article/10.3390/ijms27093833/s1.
