# Supplementary material for: Plant Defense Activation by Endophytic Metarhizium anisopliae and Beauveria bassiana Fungi Against Subterranean Termites
Source: Int J Mol Sci. 2026 Apr 25;27(9):3833. doi: 10.3390/ijms27093833 (PMC13163553; doi:10.3390/ijms27093833)
Supplement: Supplementary file 1 [file ijms-27-03833-s001.zip › ijms-4219428-supplementary.pdf]

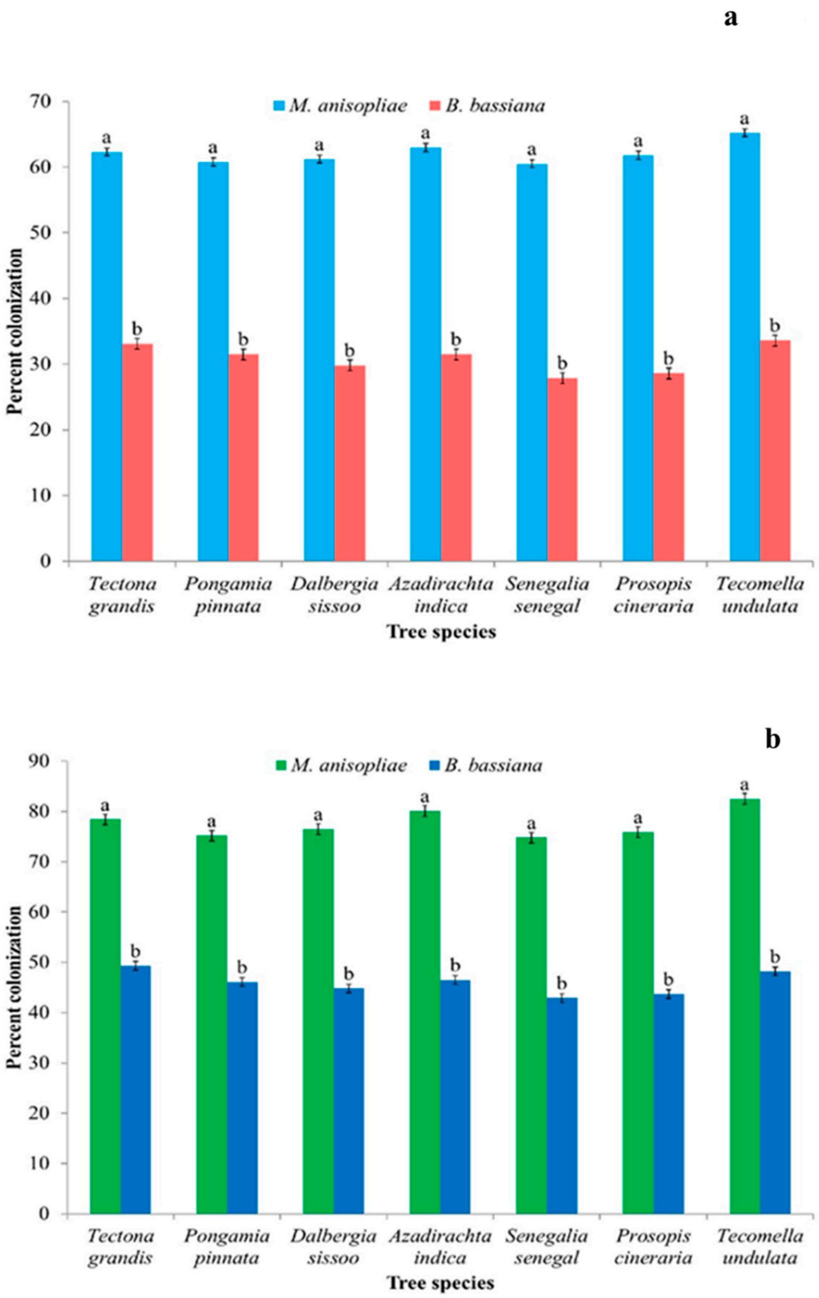

Supplementary Figure S1. Endophytic colonization (%) of *Metarhizium anisopliae* and *Beauveria bassiana* in seven forest tree species following different inoculation methods. (a) Foliar application method and (b) soil incorporation method. **Values are expressed as mean ± standard error (SE). Different lowercase letters (a,b) indicate statistically significant differences among treatments within each plant species and tissue at p ≤ 0.05.**

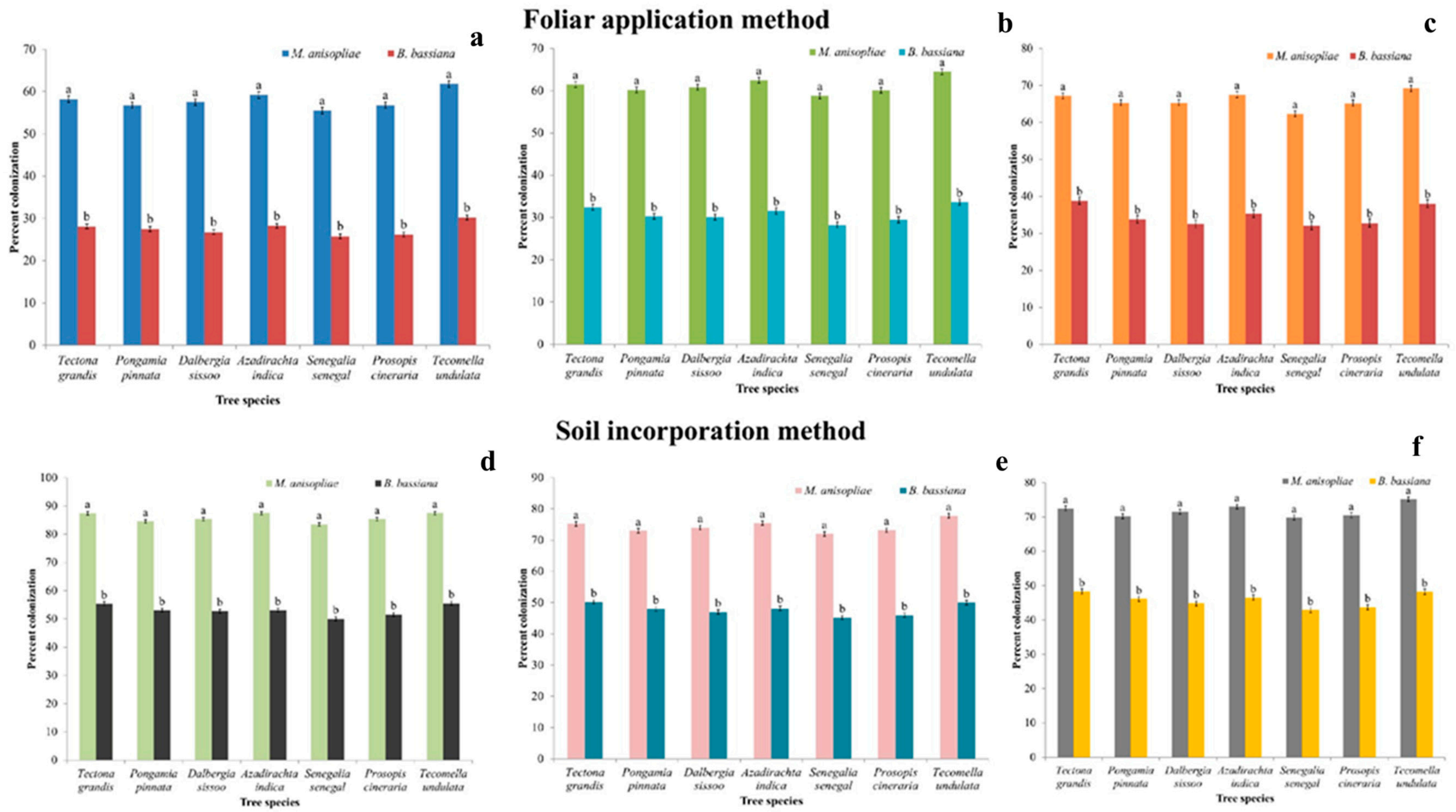

Supplementary Figure S2. Tissue-specific endophytic colonization (%) of *Metarhizium anisopliae* and *Beauveria bassiana* in seven forest tree species following different inoculation methods. (a-c) Foliar application method: (a) root, (b) stem, and (c) leaves. (d-f) Soil incorporation method: (d) root, (e) stem, and (f) leaves. **Values are expressed as mean ± standard error (SE). Different lowercase letters (a,b) indicate statistically significant differences among treatments within each plant species and tissue at $p \leq 0.05$.**

Supplementary Table S1. Variation in protein content (mg g^{-1}), phenol content (mg g^{-1}) and tannin content (mg g^{-1}) in root, stem, and leaf tissues of seven tree species seedlings in response to endophytic colonization by entomopathogenic fungi

**Tree Species**	**Treatment**	**Protein**			**Phenol**			**Tannin**		
		**Leaves (mg/g ± SE)**	**Stem (mg/g ± SE)**	**Root (mg/g ± SE)**	**Leaves (mg/g ± SE)**	**Stem (mg/g ± SE)**	**Root (mg/g ± SE)**	**Leaves (mg/g ± SE)**	**Stem (mg/g ± SE)**	**Root (mg/g ± SE)**
	M. anisopliae	2.85 ± 0.12 ^{a}	3.12 ± 0.14 ^{a}	3.45 ± 0.15 ^{a}	2.10 ± 0.12 ^{b}	1.85 ± 0.10 ^{b}	3.25 ± 0.15 ^{b}	1.65 ± 0.09 ^{b}	1.50 ± 0.08 ^{b}	2.20 ± 0.10 ^{b}
Tectona grandis	*B. bassiana*	2.62 ± 0.11 ^{b}	2.88 ± 0.13 ^{b}	3.18 ± 0.14 ^{b}	2.25 ± 0.11 ^{ab}	2.00 ± 0.09 ^{ab}	3.50 ± 0.12 ^{ab}	1.80 ± 0.08 ^{ab}	1.65 ± 0.07 ^{ab}	2.35 ± 0.09 ^{ab}
	Control	2.35 ± 0.10 ^{c}	2.61 ± 0.11 ^{c}	2.88 ± 0.12 ^{c}	1.80 ± 0.10 ^{c}	1.60 ± 0.08 ^{c}	3.00 ± 0.10 ^{c}	1.40 ± 0.07 ^{c}	1.30 ± 0.06 ^{c}	2.00 ± 0.08 ^{c}
	M. anisopliae	2.72 ± 0.11 ^{a}	2.95 ± 0.12 ^{a}	3.25 ± 0.14 ^{a}	2.50 ± 0.10 ^{a}	2.20 ± 0.11 ^{a}	3.75 ± 0.12 ^{a}	2.10 ± 0.08 ^{a}	1.90 ± 0.09 ^{a}	2.70 ± 0.10 ^{a}
Pongamia pinnata	*B. bassiana*	2.50 ± 0.10 ^{b}	2.72 ± 0.11 ^{b}	3.05 ± 0.13 ^{b}	2.35 ± 0.09 ^{ab}	2.05 ± 0.10 ^{ab}	3.60 ± 0.11 ^{ab}	2.00 ± 0.07 ^{ab}	1.80 ± 0.08 ^{ab}	2.60 ± 0.09 ^{ab}
	Control	2.28 ± 0.09 ^{c}	2.52 ± 0.10 ^{c}	2.78 ± 0.11 ^{c}	2.00 ± 0.08 ^{b}	1.85 ± 0.09 ^{b}	3.20 ± 0.10 ^{b}	1.70 ± 0.06 ^{b}	1.55 ± 0.07 ^{b}	2.30 ± 0.08 ^{b}
	M. anisopliae	2.55 ± 0.11 ^{a}	2.78 ± 0.12 ^{a}	3.10 ± 0.13 ^{a}	2.40 ± 0.11 ^{a}	2.10 ± 0.09 ^{a}	3.65 ± 0.13 ^{a}	2.00 ± 0.08 ^{a}	1.80 ± 0.08 ^{a}	2.60 ± 0.09 ^{a}
Dalbergia sissoo	*B. bassiana*	2.32 ± 0.10 ^{b}	2.55 ± 0.11 ^{b}	2.85 ± 0.12 ^{b}	2.30 ± 0.10 ^{ab}	2.00 ± 0.08 ^{ab}	3.50 ± 0.12 ^{ab}	1.90 ± 0.07 ^{ab}	1.70 ± 0.07 ^{ab}	2.50 ± 0.08 ^{ab}
	Control	1.45 ± 0.08 ^{c}	1.68 ± 0.09 ^{c}	1.98 ± 0.10 ^{c}	2.05 ± 0.09 ^{b}	1.80 ± 0.08 ^{b}	3.20 ± 0.11 ^{b}	1.65 ± 0.06 ^{b}	1.50 ± 0.06 ^{b}	2.30 ± 0.08 ^{b}
	M. anisopliae	2.88 ± 0.13 ^{a}	3.12 ± 0.14 ^{a}	3.45 ± 0.16 ^{a}	2.20 ± 0.10 ^{a}	1.95 ± 0.09 ^{a}	3.50 ± 0.12 ^{a}	1.85 ± 0.08 ^{a}	1.70 ± 0.07 ^{a}	2.45 ± 0.09 ^{a}
Azadirachta indica	*B. bassiana*	2.60 ± 0.11 ^{b}	2.85 ± 0.12 ^{b}	3.15 ± 0.14 ^{b}	2.10 ± 0.09 ^{ab}	1.85 ± 0.08 ^{ab}	3.40 ± 0.11 ^{ab}	1.75 ± 0.07 ^{ab}	1.60 ± 0.07 ^{ab}	2.35 ± 0.08 ^{ab}
	Control	2.33 ± 0.10 ^{c}	2.60 ± 0.11 ^{c}	2.88 ± 0.12 ^{c}	1.85 ± 0.08 ^{b}	1.65 ± 0.07 ^{b}	3.10 ± 0.10 ^{b}	1.50 ± 0.06 ^{b}	1.40 ± 0.06 ^{b}	2.10 ± 0.08 ^{b}
	M. anisopliae	2.50 ± 0.11 ^{a}	2.75 ± 0.12 ^{a}	3.05 ± 0.13 ^{a}	2.25 ± 0.11 ^{a}	2.00 ± 0.10 ^{a}	3.55 ± 0.12 ^{a}	1.90 ± 0.09 ^{a}	1.75 ± 0.08 ^{a}	2.55 ± 0.10 ^{a}
Senegalia senegal	*B. bassiana*	2.28 ± 0.10 ^{b}	2.52 ± 0.11 ^{b}	2.82 ± 0.12 ^{b}	2.10 ± 0.10 ^{ab}	1.90 ± 0.09 ^{ab}	3.45 ± 0.11 ^{ab}	1.80 ± 0.08 ^{ab}	1.65 ± 0.07 ^{ab}	2.45 ± 0.09 ^{ab}
	Control	1.35 ± 0.07 ^{c}	1.60 ± 0.08 ^{c}	1.90 ± 0.09 ^{c}	1.95 ± 0.09 ^{b}	1.75 ± 0.08 ^{b}	3.15 ± 0.10 ^{b}	1.55 ± 0.07 ^{b}	1.45 ± 0.06 ^{b}	2.20 ± 0.08 ^{b}
	M. anisopliae	2.75 ± 0.11 ^{a}	3.00 ± 0.12 ^{a}	3.33 ± 0.14 ^{a}	2.15 ± 0.10 ^{a}	1.95 ± 0.09 ^{a}	3.45 ± 0.11 ^{a}	1.80 ± 0.08 ^{a}	1.65 ± 0.07 ^{a}	2.40 ± 0.09 ^{a}
Prosopis cineraria	*B. bassiana*	2.52 ± 0.10 ^{b}	2.75 ± 0.11 ^{b}	3.08 ± 0.13 ^{b}	2.05 ± 0.09 ^{ab}	1.85 ± 0.08 ^{ab}	3.35 ± 0.10 ^{ab}	1.70 ± 0.07 ^{ab}	1.55 ± 0.06 ^{ab}	2.30 ± 0.08 ^{ab}
	Control	2.30 ± 0.09 ^{c}	2.55 ± 0.10 ^{c}	2.82 ± 0.12 ^{c}	1.90 ± 0.08 ^{b}	1.70 ± 0.07 ^{b}	3.10 ± 0.09 ^{b}	1.45 ± 0.06 ^{b}	1.35 ± 0.06 ^{b}	2.05 ± 0.07 ^{b}
	M. anisopliae	2.92 ± 0.13 ^{a}	3.18 ± 0.14 ^{a}	3.50 ± 0.16 ^{a}	2.30 ± 0.11 ^{a}	2.05 ± 0.09 ^{a}	3.60 ± 0.12 ^{a}	1.95 ± 0.09 ^{a}	1.80 ± 0.08 ^{a}	2.65 ± 0.10 ^{a}
Tecomella undulata	*B. bassiana*	2.62 ± 0.11 ^{b}	2.88 ± 0.12 ^{b}	3.18 ± 0.14 ^{b}	2.20 ± 0.10 ^{ab}	1.95 ± 0.08 ^{ab}	3.50 ± 0.11 ^{ab}	1.85 ± 0.08 ^{ab}	1.70 ± 0.07 ^{ab}	2.50 ± 0.09 ^{ab}
	Control	2.35 ± 0.10 ^{c}	2.62 ± 0.11 ^{c}	2.88 ± 0.12 ^{c}	2.00 ± 0.09 ^{b}	1.80 ± 0.08 ^{b}	3.20 ± 0.10 ^{b}	1.60 ± 0.07 ^{b}	1.50 ± 0.06 ^{b}	2.25 ± 0.08 ^{b}
Source of variation	Degree of freedom	F-probability	P- value	LSD	F-probability	P- value	LSD	F-probability	P- value	LSD
Seedlings (A)	6	32.76	<0.001	0.16	8.73	<0.001	0.14	10.65	<0.001	0.14
EPF (B)	2	165.96	<0.001	0.18	66.51	<0.001	0.15	98.56	<0.001	0.16
Seedlings part (C)	2	63.14	<0.001	0.17	145.26	<0.001	0.14	101.45	<0.001	0.17
A×B	12	5.57	<0.001	0.16	2.21	0.015	0.14	3.11	<0.001	0.16
A×C	12	2.06	0.066	0.18	1.10	0.370	0.14	10.23	0.568	0.15
B×C	4	0.91	0.406	0.17	3.33	0.012	0.13	0.58	0.023	0.16
A×B×C	24	1.38	0.193	0.16	1.16	0.295	0.13	2.34	0.019	0.15

LSD= Least significant difference

Supplementary Table S2. Catalase, Ascorbate oxidase, Phenylalanine Ammonia-Lyase and Tyrosine Ammonia-Lyase activity (U/mg protein ± SE) in root, stem, and leaves of seven tree species seedlings under fungal inoculation and control

Tree Species	Treatment	Catalase			Ascorbate oxidase			Phenylalanine Ammonia-Lyase			Tyrosine Ammonia-Lyase		
		Leaves (U/mL ± SE)	Stem (U/mL ± SE)	Root (U/mL ± SE)	Leaves (U/mL ± SE)	Stem (U/mL ± SE)	Root (U/mL ± SE)	Leaves (U/mL ± SE)	Stem (U/mL ± SE)	Root (U/mL ± SE)	Leaves (U/mL ± SE)	Stem (U/mL ± SE)	Root (U/mL ± SE)
Tectona grandis	*M. anisopliae*	210.45 ± 3.21 ^{a}	198.32 ± 2.95 ^{b}	220.18 ± 3.54 ^{a}	100.12 ± 1.60 ^{a}	98.45 ± 1.52 ^{a}	94.36 ± 1.48 ^{a}	152.12 ± 2.15 ^{a}	158.45 ± 2.25 ^{a}	162.78 ± 2.30 ^{a}	185.12 ± 2.40 ^{a}	190.45 ± 2.50 ^{a}	195.78 ± 2.55 ^{a}
	B. bassiana	175.67 ± 2.83 ^{b}	160.44 ± 2.62 ^{c}	185.29 ± 3.03 ^{b}	80.11 ± 1.34 ^{b}	75.23 ± 1.28 ^{b}	68.44 ± 1.22 ^{b}	123.44 ± 1.88 ^{b}	130.12 ± 1.95 ^{b}	135.78 ± 2.05 ^{b}	150.12 ± 2.00 ^{b}	160.45 ± 2.10 ^{b}	165.78 ± 2.15 ^{b}
	Control	85.23 ± 1.91 ^{d}	78.11 ± 1.84 ^{d}	90.45 ± 2.02 ^{d}	36.78 ± 0.88 ^{d}	33.12 ± 0.80 ^{d}	28.45 ± 0.72 ^{d}	78.45 ± 1.12 ^{c}	81.22 ± 1.18 ^{c}	83.11 ± 1.20 ^{c}	92.12 ± 1.20 ^{c}	95.45 ± 1.25 ^{c}	98.78 ± 1.30 ^{c}
Pongamia pinnata	*M. anisopliae*	198.12 ± 3.05 ^{a}	205.36 ± 3.21 ^{a}	212.48 ± 3.42 ^{a}	97.22 ± 1.55 ^{a}	92.36 ± 1.44 ^{a}	95.48 ± 1.50 ^{a}	158.23 ± 2.22 ^{a}	162.56 ± 2.30 ^{a}	166.12 ± 2.35 ^{a}	188.12 ± 2.45 ^{a}	192.45 ± 2.50 ^{a}	196.78 ± 2.55 ^{a}
	B. bassiana	160.23 ± 2.63 ^{b}	155.11 ± 2.54 ^{b}	170.36 ± 2.84 ^{b}	72.18 ± 1.30 ^{b}	65.12 ± 1.20 ^{b}	70.34 ± 1.28 ^{b}	118.34 ± 1.75 ^{b}	125.44 ± 1.88 ^{b}	128.78 ± 1.92 ^{b}	152.12 ± 2.05 ^{b}	158.45 ± 2.10 ^{b}	162.78 ± 2.15 ^{b}
	Control	80.54 ± 1.82 ^{d}	75.33 ± 1.71 ^{d}	82.18 ± 1.90 ^{d}	31.78 ± 0.78 ^{d}	30.45 ± 0.75 ^{d}	34.22 ± 0.82 ^{d}	82.12 ± 1.20 ^{c}	85.45 ± 1.25 ^{c}	88.78 ± 1.30 ^{c}	95.12 ± 1.25 ^{c}	98.45 ± 1.30 ^{c}	101.78 ± 1.35 ^{c}
Dalbergia sissoo	*M. anisopliae*	225.67 ± 3.52 ^{a}	240.12 ± 3.83 ^{a}	230.48 ± 3.61 ^{a}	98.44 ± 1.52 ^{a}	100.12 ± 1.55 ^{a}	102.33 ± 1.60 ^{a}	162.78 ± 2.35 ^{a}	166.12 ± 2.40 ^{a}	170.45 ± 2.45 ^{a}	192.12 ± 2.50 ^{a}	195.45 ± 2.55 ^{a}	198.78 ± 2.60 ^{a}
	B. bassiana	185.29 ± 3.02 ^{b}	192.11 ± 3.22 ^{b}	180.56 ± 2.94 ^{b}	80.78 ± 1.28 ^{b}	82.45 ± 1.35 ^{b}	87.12 ± 1.40 ^{b}	128.45 ± 1.92 ^{b}	135.78 ± 2.05 ^{b}	138.12 ± 2.10 ^{b}	158.12 ± 2.10 ^{b}	162.45 ± 2.15 ^{b}	165.78 ± 2.20 ^{b}
	Control	60.34 ± 1.52 ^{d}	55.12 ± 1.44 ^{d}	65.23 ± 1.61 ^{d}	44.00 ± 0.90 ^{d}	42.36 ± 0.88 ^{d}	40.12 ± 0.85 ^{d}	84.12 ± 1.30 ^{c}	88.45 ± 1.35 ^{c}	91.78 ± 1.40 ^{c}	98.12 ± 1.30 ^{c}	101.45 ± 1.35 ^{c}	104.78 ± 1.40 ^{c}
Azadirachta indica	*M. anisopliae*	198.76 ± 3.01 ^{a}	210.25 ± 3.33 ^{b}	205.48 ± 3.21 ^{a}	96.22 ± 1.53 ^{a}	90.12 ± 1.40 ^{a}	93.48 ± 1.48 ^{a}	155.34 ± 2.25 ^{a}	158.78 ± 2.30 ^{a}	162.12 ± 2.35 ^{a}	185.78 ± 2.42 ^{a}	190.12 ± 2.50 ^{a}	193.45 ± 2.55 ^{a}
	B. bassiana	160.45 ± 2.62 ^{b}	175.32 ± 2.81 ^{b}	170.44 ± 2.72 ^{b}	68.44 ± 1.28 ^{b}	60.34 ± 1.15 ^{b}	65.12 ± 1.20 ^{b}	123.12 ± 1.88 ^{b}	128.45 ± 1.95 ^{b}	130.78 ± 2.00 ^{b}	150.78 ± 2.05 ^{b}	155.12 ± 2.10 ^{b}	158.45 ± 2.15 ^{b}
	Control	82.36 ± 1.90 ^{d}	78.23 ± 1.84 ^{d}	85.12 ± 2.01 ^{d}	33.22 ± 0.78 ^{d}	28.12 ± 0.72 ^{d}	30.45 ± 0.75 ^{d}	79.78 ± 1.20 ^{c}	82.12 ± 1.25 ^{c}	84.45 ± 1.28 ^{c}	92.78 ± 1.25 ^{c}	95.12 ± 1.30 ^{c}	98.45 ± 1.35 ^{c}
Senegalia senegal	*M. anisopliae*	220.12 ± 3.41 ^{a}	230.36 ± 3.62 ^{a}	215.48 ± 3.33 ^{a}	98.45 ± 1.55 ^{a}	95.78 ± 1.50 ^{a}	97.22 ± 1.53 ^{a}	160.12 ± 2.30 ^{a}	163.45 ± 2.35 ^{a}	166.78 ± 2.40 ^{a}	188.45 ± 2.45 ^{a}	192.78 ± 2.50 ^{a}	196.12 ± 2.55 ^{a}
	B. bassiana	170.45 ± 2.93 ^{b}	165.12 ± 2.81 ^{b}	180.34 ± 3.02 ^{b}	80.11 ± 1.40 ^{b}	72.12 ± 1.28 ^{b}	77.34 ± 1.34 ^{b}	125.12 ± 1.92 ^{b}	130.45 ± 1.98 ^{b}	132.78 ± 2.05 ^{b}	152.45 ± 2.05 ^{b}	158.78 ± 2.10 ^{b}	162.12 ± 2.15 ^{b}
	Control	58.23 ± 1.52 ^{d}	55.48 ± 1.44 ^{d}	60.12 ± 1.55 ^{d}	33.45 ± 0.80 ^{d}	31.78 ± 0.78 ^{d}	35.12 ± 0.82 ^{d}	82.12 ± 1.25 ^{c}	85.45 ± 1.30 ^{c}	88.78 ± 1.35 ^{c}	95.45 ± 1.30 ^{c}	98.78 ± 1.35 ^{c}	102.12 ± 1.40 ^{c}
Prosopis cineraria	*M. anisopliae*	198.33 ± 3.02 ^{a}	205.48 ± 3.21 ^{a}	210.12 ± 3.33 ^{a}	93.12 ± 1.50 ^{a}	88.45 ± 1.42 ^{a}	91.33 ± 1.46 ^{a}	152.78 ± 2.20 ^{a}	155.12 ± 2.25 ^{a}	158.45 ± 2.30 ^{a}	185.12 ± 2.40 ^{a}	190.45 ± 2.50 ^{a}	195.78 ± 2.55 ^{a}
	B. bassiana	160.11 ± 2.63 ^{b}	155.29 ± 2.54 ^{b}	170.36 ± 2.82 ^{b}	65.78 ± 1.28 ^{b}	55.12 ± 1.10 ^{b}	62.44 ± 1.22 ^{b}	110.12 ± 1.75 ^{b}	118.45 ± 1.88 ^{b}	120.78 ± 1.92 ^{b}	150.12 ± 2.00 ^{b}	155.45 ± 2.10 ^{b}	160.78 ± 2.15 ^{b}
	Control	80.45 ± 1.82 ^{d}	75.48 ± 1.71 ^{d}	82.36 ± 1.90 ^{d}	27.12 ± 0.70 ^{d}	25.78 ± 0.70 ^{d}	28.44 ± 0.72 ^{d}	78.12 ± 1.20 ^{c}	81.45 ± 1.25 c	84.78 ± 1.28 ^{c}	92.12 ± 1.25 ^{c}	95.45 ± 1.30 ^{c}	98.78 ± 1.35 ^{c}
Tecomella undulata	*M. anisopliae*	250.12 ± 4.01 ^{a}	263.48 ± 4.22 ^{a}	255.33 ± 4.12 ^{a}	100.78 ± 1.55 ^{a}	101.22 ± 1.58 ^{a}	102.00 ± 1.60 ^{a}	163.12 ± 2.35 ^{a}	166.45 ± 2.40 ^{a}	170.78 ± 2.45 ^{a}	192.12 ± 2.50 ^{a}	195.45 ± 2.55 ^{a}	198.78 ± 2.60 ^{a}
	B. bassiana	190.23 ± 3.21 ^{b}	197.12 ± 3.33 ^{b}	185.48 ± 3.01 ^{b}	82.44 ± 1.30 ^{b}	85.12 ± 1.34 ^{b}	87.00 ± 1.36 ^{b}	135.12 ± 2.05 ^{b}	138.45 ± 2.10 ^{b}	140.78 ± 2.15 ^{b}	160.12 ± 2.10 ^{b}	165.45 ± 2.15 ^{b}	168.78 ± 2.20 ^{b}
	Control	95.36 ± 2.01 ^{d}	90.12 ± 1.92 ^{d}	98.45 ± 2.10 ^{d}	44.00 ± 0.90 ^{d}	40.12 ± 0.85 ^{d}	42.33 ± 0.88 ^{d}	88.12 ± 1.35 ^{c}	91.45 ± 1.40 ^{c}	94.78 ± 1.45 ^{c}	98.12 ± 1.35 ^{c}	101.45 ± 1.40 ^{c}	104.78 ± 1.45 ^{c}

Source of variation	Degree of freedom	F-probability	P- value	LSD	F-probability	P- value	LSD	F-probability	P- value	LSD	F-probability	P- value	LSD
Seedlings (A)	6	161.35	<0.001	0.18	226.64	<0.001	0.1236	59.94	<0.001	2.35	18.27	<0.001	0.89
EPF (B)	2	14654.10	<0.001	0.16	14124.80	<0.001	0.1036	8245.75	<0.001	3.12	12118.52	<0.001	0.46
Seedlings part (C)	2	12.20	<0.001	0.15	37.18	<0.001	0.0936	100.84	<0.001	2.85	75.71	<0.001	1.23
A×B	12	59.35	<0.001	0.2	20.18	<0.001	0.1236	5.04	<0.001	2.32	2.12	<0.001	0.96
A×C	12	6.27	<0.001	0.19	12.10	<0.001	0.1036	0.95	0.5036	3.2	1.50	0.131607	0.43
B×C	4	4.67	<0.001	0.17	1.56	0.1887	0.0936	1.65	0.1667	2.88	3.56	<0.001	1.31
A×B×C	24	4.28	<0.001	0.16	3.38	<0.001	0.14	1.12	0.3273	2.42	1.23	0.23085	0.99

LSD= Least significant difference

Supplementary Table S3. Termite mortality and mycosis (%) following feeding on EPF-treated seedlings

Tree Species	Treatment	Mortality Root (%)	Mortality Stem (%)	Mycosis Root (%)	Mycosis Stem (%)	F-value	p-value	LSD (p=0.05)
Tectona grandis	*M. anisopliae*	78.2 ± 1.9 a	82.1 ± 1.7 a	61.5 ± 1.6 a	65.8 ± 1.5 a	108.6	<0.001	4.2
	B. bassiana	66.4 ± 2.1 b	71.5 ± 1.9 b	52.3 ± 1.8 b	56.7 ± 1.7 b			
	Control	8.5 ± 0.8 c	9.2 ± 0.9 c	0.0 c	0.0 c			
Pongamia pinnata	*M. anisopliae*	80.3 ± 1.8 a	84.5 ± 1.6 a	63.8 ± 1.5 a	68.2 ± 1.4 a	86.76	<0.001	2.5
	B. bassiana	70.2 ± 2.0 b	74.3 ± 1.8 b	55.2 ± 1.7 b	59.8 ± 1.6 b			
	Control	7.8 ± 0.7 c	8.6 ± 0.8 c	0.0 c	0.0 c			
Dalbergia sissoo	*M. anisopliae*	79.1 ± 1.7 a	83.2 ± 1.5 a	62.7 ± 1.5 a	66.9 ± 1.4 a	115.46	<0.001	4.22
	B. bassiana	68.5 ± 1.9 b	72.4 ± 1.7 b	53.8 ± 1.7 b	57.9 ± 1.6 b			
	Control	6.9 ± 0.7 c	7.5 ± 0.8 c	0.0 c	0.0 c			
Azadirachta indica	*M. anisopliae*	85.2 ± 1.5 a	88.7 ± 1.4 a	68.9 ± 1.4 a	72.5 ± 1.3 a	101.66	<0.001	1.56
	B. bassiana	77.1 ± 1.8 b	81.0 ± 1.6 b	61.0 ± 1.6 b	65.1 ± 1.5 b			
	Control	8.2 ± 0.8 c	9.0 ± 0.9 c	0.0 c	0.0 c			
Senegalia senegal	*M. anisopliae*	73.4 ± 1.9 a	77.8 ± 1.6 a	57.2 ± 1.5 a	61.3 ± 1.5 a	92.56	<0.001	3.11
	B. bassiana	61.2 ± 2.0 b	66.5 ± 1.9 b	48.9 ± 1.7 b	52.7 ± 1.6 b			
	Control	7.1 ± 0.7 c	7.8 ± 0.8 c	0.0 c	0.0 c			
Prosopis cineraria	*M. anisopliae*	87.3 ± 1.4 a	90.2 ± 1.3 a	70.1 ± 1.3 a	74.0 ± 1.2 a	124.72	<0.001	2.05
	B. bassiana	78.4 ± 1.7 b	82.6 ± 1.5 b	63.2 ± 1.5 b	66.8 ± 1.4 b			
	Control	6.5 ± 0.6 c	7.2 ± 0.7 c	0.0 c	0.0 c			
Tecomella undulata	*M. anisopliae*	81.5 ± 1.6 a	85.0 ± 1.5 a	65.0 ± 1.4 a	69.4 ± 1.3 a	109.48	<0.001	2.36
	B. bassiana	70.9 ± 1.8 b	75.8 ± 1.7 b	56.8 ± 1.6 b	60.5 ± 1.5 b			
	Control	8.0 ± 0.8 c	8.7 ± 0.9 c	0.0 c	0.0 c			

LSD= least significant difference
